# Phosphorylation of auxin signaling repressor IAA8 by heat-responsive MPKs causes defective flower development

**DOI:** 10.1093/plphys/kiae470

**Published:** 2024-09-06

**Authors:** Sun Ho Kim, Shah Hussain, Huyen Trang Thi Pham, Ulhas Sopanrao Kadam, Sunghwa Bahk, Zakiyah Ramadany, Jeongwoo Lee, Young Hun Song, Kyun Oh Lee, Jong Chan Hong, Woo Sik Chung

**Affiliations:** Division of Applied Life Science (BK21 Four program), Plant Molecular Biology and Biotechnology Research Center, Gyeongsang National University, Jinju 52828, Republic of Korea; Division of Applied Life Science (BK21 Four program), Plant Molecular Biology and Biotechnology Research Center, Gyeongsang National University, Jinju 52828, Republic of Korea; Division of Applied Life Science (BK21 Four program), Plant Molecular Biology and Biotechnology Research Center, Gyeongsang National University, Jinju 52828, Republic of Korea; Division of Applied Life Science (BK21 Four program), Plant Molecular Biology and Biotechnology Research Center, Gyeongsang National University, Jinju 52828, Republic of Korea; Division of Applied Life Science (BK21 Four program), Plant Molecular Biology and Biotechnology Research Center, Gyeongsang National University, Jinju 52828, Republic of Korea; Division of Applied Life Science (BK21 Four program), Plant Molecular Biology and Biotechnology Research Center, Gyeongsang National University, Jinju 52828, Republic of Korea; Division of Applied Life Science (BK21 Four program), Plant Molecular Biology and Biotechnology Research Center, Gyeongsang National University, Jinju 52828, Republic of Korea; Depatment of Applied Biology and Chemistry, Seoul National University, Seoul 08826, Republic of Korea; Division of Applied Life Science (BK21 Four program), Plant Molecular Biology and Biotechnology Research Center, Gyeongsang National University, Jinju 52828, Republic of Korea; Division of Applied Life Science (BK21 Four program), Plant Molecular Biology and Biotechnology Research Center, Gyeongsang National University, Jinju 52828, Republic of Korea; Division of Applied Life Science (BK21 Four program), Plant Molecular Biology and Biotechnology Research Center, Gyeongsang National University, Jinju 52828, Republic of Korea

## Abstract

Heat stress is a substantial and imminent threat to plant growth and development. Understanding its adverse effects on plant development at the molecular level is crucial for sustainable agriculture. However, the molecular mechanism underlying how heat stress causes developmental defects in flowers remains poorly understood. Here, we identified Indole-3-Acetic Acid 8 (IAA8), a repressor of auxin signaling, as a substrate of mitogen-activated protein kinases (MPKs) in *Arabidopsis thaliana*, and found that MPK-mediated phosphorylation of IAA8 inhibits flower development. MPKs phosphorylated three residues of IAA8: S74, T77, and S135. Interestingly, transgenic plants overexpressing a phospho-mimicking mutant of IAA8 (IAA8^DDD^ OX) exhibited defective flower development due to high IAA8 levels. Furthermore, MPK-mediated phosphorylation inhibited IAA8 polyubiquitination, thereby significantly increasing its stability. Additionally, the expression of key transcription factors involved in flower development, such as *bZIP* and *MYB* genes, was significantly perturbed in the IAA8^DDD^ OX plants. Collectively, our study demonstrates that heat stress inhibits flower development by perturbing the expression of flower development genes through the MPK-mediated phosphorylation of IAA8, suggesting that Aux/IAA phosphorylation enables plants to fine-tune their development in response to environmental stress.

## Teaser

Heat-responsive MPKs phosphorylate and stabilize an auxin signaling repressor, thereby causing defective flower development by perturbing the expression of flower development genes in Arabidopsis.

## Introduction

Environmental stresses negatively impact plant growth, development, and yield (Sakata et al. 2010; Su et al. 2013; An et al. 2017). Among them, heat stress acts as a significant threat to plant growth and reproductive development by reducing endogenous auxin levels (Sakata et al. 2010). Heat stress-induced defective flower development is characterized by abnormal anthers, reduced pollen viability, smaller filament, and aborted ovules, resulting in nonviable seeds (Sakata et al. 2010; Folsom et al. 2014; Chen et al. 2016; Begcy and Dresselhaus 2018). These abnormal phenotypes have been extensively studied in various plant species (Saini et al. 1983; Ahmed et al. 1992; Peet et al. 1998; Sakata and Higashitani 2008). Despite the well-known relationship between heat stress and repression of auxin, the underlying molecular mechanism remains largely unknown.

Auxin plays an important role in regulating numerous physiological and developmental processes, including flower development (Teale et al. 2006; Cheng and Zhao 2007; Roychoudhry and Kepinski 2022). In plants, the auxin signaling pathway is conserved (Guilfoyle and Hagen 2007; Mockaitis and Estelle 2008; Yu et al. 2022) and involves three key components: auxin co-receptors (TRANSPORT INHIBITOR RESPONSE 1/AUXIN SIGNALING F-BOX PROTEINs [TIR1/AFBs]), transcriptional repressors (AUXIN/INDOLE-3-ACETIC ACIDs [Aux/IAAs]), and transcription factors (TFs) such as AUXIN RESPONSE FACTORs (ARFs). At low levels of auxin, Aux/IAAs physically interact with ARFs, thereby inhibiting their transcriptional activity. Conversely, when auxin levels increase, auxin triggers the interaction between TIR1/AFBs and Aux/IAAs, resulting in the ubiquitin-mediated degradation of Aux/IAAs. This degradation leads to the release of ARFs, enabling them to initiate the transcription of auxin-responsive genes (Gray et al. 2001; Tiwari et al. 2001; Kepinski and Leyser 2002; Weijers et al. 2005). Moreover, extensive studies involving loss- or gain-of-function mutants have elucidated the physiological functions of ARFs and Aux/IAAs. Some mutants that inhibit auxin signaling exhibit defective flower development. For example, the *arf6*/*arf8* double knockout mutant displays flowers with defects attributable to decreased jasmonic acid (JA) levels (Nagpal et al. 2005; Tabata et al. 2010). In addition, the Indole-3-Acetic Acid 8 (IAA8) gain-of-function mutant exhibited developmental defects in flowers due to decreased JA levels (Wang et al. 2013). JA biosynthesis mediated by auxin signaling is reported to be required for normal flower developments, including floral organ formation, flower opening, pollen maturation, and anther dehiscence at the middle stages of flower development (Ishiguro et al. 2001; Nagpal et al. 2005; Tabata et al. 2010). Meanwhile, the gain-of-function mutant of *massugu2-1* (*msg2-1*)/*iaa19* showed abnormal pistils and stamens at the late stages of flower development (Tashiro et al. 2009).

Numerous transcriptional factors (TFs) are also known to regulate flower development. For example, the basic leucine zipper (bZIP) TFs, including bZIP28 and bZIP60, play a positive role in flower development by participating in the unfolded protein response (UPR) pathway under heat stress (Zhang et al. 2017; Liu et al. 2022b). The double mutant of *bzip28*/*bzip60*, which is known to be UPR-deficient mutant, exhibits heat stress sensitivity, reduced fertility, and shortened siliques (Zhang et al. 2017). Furthermore, optimal expressions of flower-specific R2R3 type MYB TFs, *MYB21*, *MYB24*, and *MYB104* are crucial for flower development. In fact, loss-of-function mutants and transgenic plants overexpressing these genes have shown abnormal flower development (Cheng et al. 2009; Mandaokar and Browse 2009; Reeves et al. 2012).

Several studies have suggested the involvement of MPK cascades in auxin signaling. For instance, an MPK cascade activated by a plant-specific MAPKKK causes the transcriptional inhibition of the early auxin-responsive gene *GH3* (Kovtun et al. 1998, 2000). Negative regulation of auxin signaling by MPK12 and positive regulation by the specific phosphatase inactivating MPK12, IBR5, have been reported in Arabidopsis (*Arabidopsis thaliana*) (Lee et al. 2009). Additionally, an MKK7-MPK6 module regulates shoot branching by modulating polar auxin transport (Jia et al. 2016). However, the molecular mechanisms by which MPK cascades suppress the auxin signaling pathway are still poorly understood and remain unclear. Although MPK activities are known to be rapidly increased by heat stress, the specific biological roles of these cascades under such conditions have not been extensively investigated.

Previous studies have suggested that IAA8 may be a potential substrate of MPKs, as indicated by protein microarray analysis (Popescu et al. 2009) and in vitro kinase assays (Kim et al. 2022). However, direct experimental evidence clarifying the physiological roles of IAA8 phosphorylation by MPKs has not yet been elucidated. Therefore, our study focused on directly assessing the phosphorylation of IAA8 by MPKs, and exploring the physiological roles of this modification. We constructed transgenic plants overexpressing a nonphosphorylatable mutant (IAA8^AAA^ OX) and a phospho-mimicking mutant (IAA8^DDD^ OX). Surprisingly, we observed that the IAA8^DDD^ OX plants exhibited defective flower development, characterized by shorter floral organs and reduced seed production. Furthermore, we found that MPK-mediated phosphorylation of IAA8 leads to its stabilization by inhibiting polyubiquitination. This modification also resulted in perturbed transcript levels of flower-specific TF genes, such as *bZIPs* and *MYBs*, in flowers of IAA8^DDD^ OX plants. These findings suggest that the phosphorylation of IAA8 contributes to defective flower development in response to heat stress.

## Results

### Heat stress suppresses auxin signaling through MPK cascades in flower

Heat stress has been reported to suppress flower development through the reduction of auxin biosynthesis and signaling (Sakata et al. 2010). This reduction in auxin biosynthesis is caused by the transcriptional down-regulation of *YUCCA* genes (Sakata et al. 2010). Therefore, the decrease of auxin signaling is believed to result from the reduced auxin levels. However, it is a possibility that the decrease of auxin signaling by heat stress could be caused by unknown pathways. To investigate this possibility, we analyzed *β-glucuronidase* (GUS) activity using an auxin reporter plant, *DR5::GUS*, which expresses GUS driven by the control of the *DR5* promoter. Consistent with previous reports, GUS activities were primarily detected in the stamen filaments and anthers of flowers under normal conditions, but this GUS activity was significantly reduced by heat stress (Supplementary Fig. S1). Since MPK cascades have been reported to suppress auxin signaling (Kovtun et al. 1998, 2000; Jonak et al. 2002; Kim et al. 2022), we hypothesized that auxin signaling could be inhibited by a heat-responsive MPK cascade. To assess this hypothesis, we observed the effects of an MKK inhibitor, U0126, on auxin signaling after the treatment with heat stress. As a result, we found that the heat-mediated inhibition of GUS activity in floral organs was highly suppressed in the presence of U0126 (Supplementary Fig. S1), indicating that the suppression of auxin signaling under heat stress is mediated by an MPK cascade.

### IAA8 is phosphorylated by the heat-responsive MPKs

Several *Aux/IAA* gain-of-function mutants exhibit typical auxin-defective phenotypes due to the constitutive suppression of auxin signaling (Reed 2001). Among these mutants, the IAA8 gain-of-function mutant showed defective floral organ development and decreased fertilization (Wang et al. 2013). Since IAA8 has been reported as a putative substrate of MPKs (Popescu et al. 2009; Kim et al. 2022), we postulated that an MPK-IAA8 module is negatively involved in flower development under heat stress by inhibiting auxin signaling.

**Figure 1.**
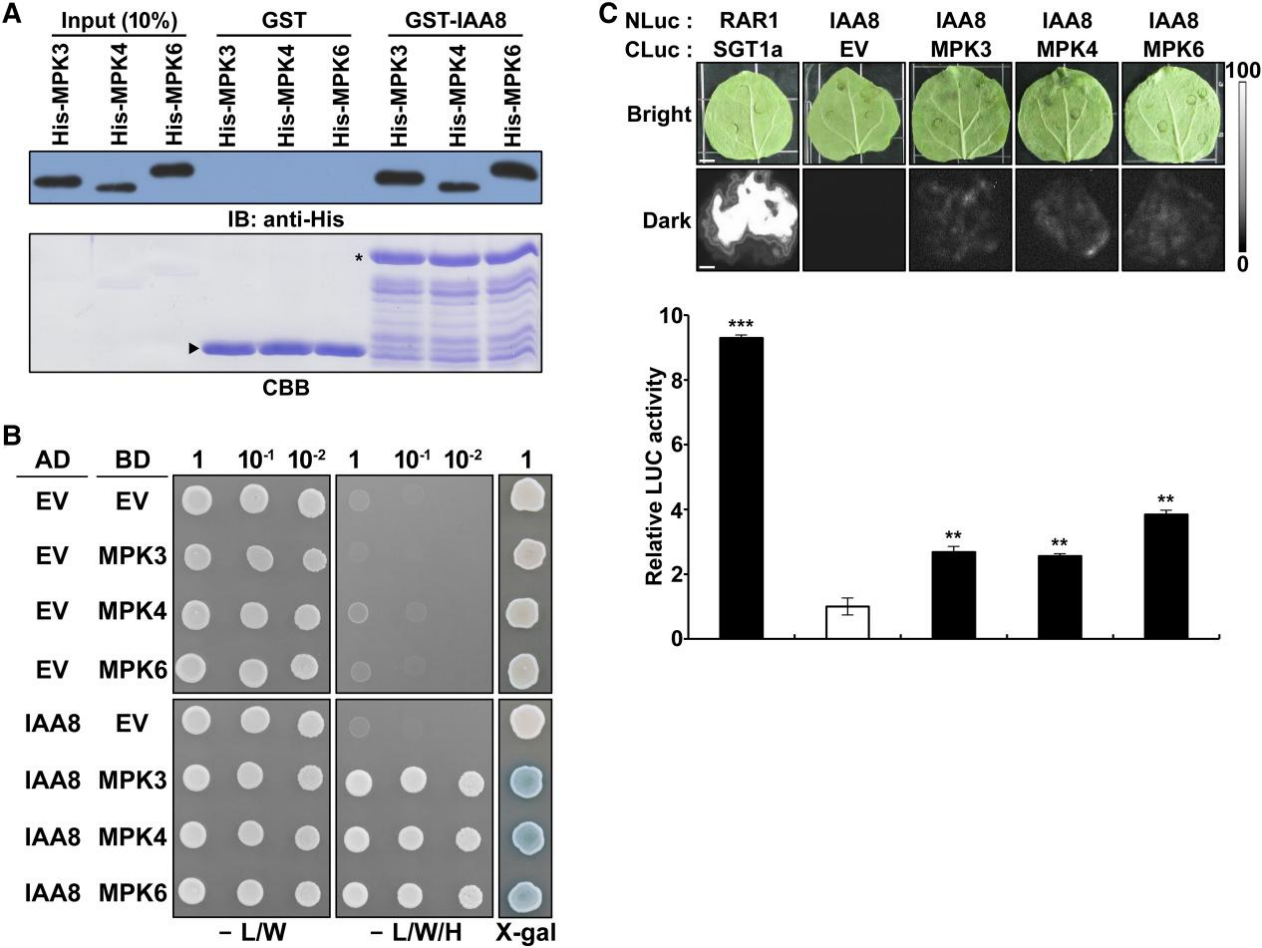
IAA8 interacts with MPKs in vitro and in vivo. **A)** A pull-down assay shows the in vitro interactions of GST-IAA8 with His-MPKs. Glutathione-Sepharose-4B resin precipitated GST-fused proteins. Immunoblotting (IB) with anti-His antibodies was performed to detect input His-MPKs and His-MPKs pulled down by GST or GST-IAA8 (upper panel). The SDS-PAGE gel was stained by CBB (lower panel). The arrowhead and star indicate GST and GST-IAA8, respectively. **B)** Yeast two-hybrid analysis demonstrates the interaction of IAA8 with MPK3, MPK4, or MPK6. AD and BD plasmids were co-transformed into yeast strain pJ69-4A. Serial dilutions of transformants were plated on a selective SD medium lacking Leu and Trp (-L/W) and on an SD medium lacking Leu, Trp, and His (-L/W/H). An X-gal assay for β-galactosidase activity (X-gal) is also shown, where blue coloration indicates high β-galactosidase activity, triggering activation of the *LacZ* reporter. **C)** LUC complementation imaging assay displaying the interaction between IAA8 and MPKs in planta. The upper panel shows bright field (bright) and luminescence (dark) images of *N. benthamiana* leaves co-infiltrated with *Agrobacterium* strains harboring various combinations of *NLuc*- and *CLuc*-fusion constructs. The *SGT1a*-*NLuc*/*CLuc*-*RAR1* combination was utilized as a positive control. EV denotes the CLuc empty vector. Imaging occurred 3 d post-infiltration. The lower panel presents the quantification of LUC activity in leaves from the upper panel, with luminescence intensities measured relative to the *IAA8*-*NLuc*/*CLuc* (EV) combination. The bars indicate the mean ± SD (*n* = 3). Significant differences were determined with the Student's *t*-test (**, *P* < 0.01; ***, *P* < 0.001). The scale bar represents 0.5 cm. The black-and-white color bar indicates the range of luminescence intensity.

To verify whether IAA8 is indeed a substrate of MPKs, we assessed the protein–protein interactions between IAA8 and MPKs using in vitro pull-down assay (Fig. 1A). Immunoblots showed that GST-IAA8 pulled down His-MPK3, His-MPK4, and His-MPK6, while GST could not. This result was further confirmed by a reverse pull-down assay (Supplementary Fig. S2), indicating that IAA8 physically interacts with MPK3, MPK4, and MPK6. To further confirm these interactions, we tested the protein–protein interaction between IAA8 and MPKs using a yeast two-hybrid assay (Fig. 1B). As a result, yeast cells co-expressing IAA8 and MPK3, MPK4, or MPK6 grew on a selective medium lacking histidine and exhibited β-galactosidase activity, directly confirming interactions of IAA8 with MPK3, MPK4, and MPK6 within yeast cells.

To estimate in planta interaction of IAA8 with MPKs, we conducted luciferase (LUC) complementation imaging (LCI) assays in *Nicotiana benthamiana*. LUC activity was strongly observed in a positive control co-expressing *SGT1a*-*NLuc* and *CLuc*-*RAR1* (Chen et al. 2008) (Fig. 1C). In contrast, no LUC activities were detected in leaves co-expressing *IAA8*-*NLuc* with the *CLuc* vector (EV) or the *NLuc* vector (EV) with combinations of *CLuc*-*MPK3*, *-MPK4*, and *-MPK6* (Fig. 1C and Supplementary Fig. S3). Significantly enhanced LUC activities were detected in leaves co-expressing *IAA8*-*NLuc* with *CLuc*-*MPK3*, *-MPK4*, and *-MPK6*, demonstrating that IAA8 interacts with MPK3, MPK4, and MPK6 in planta.

**Figure 2.**
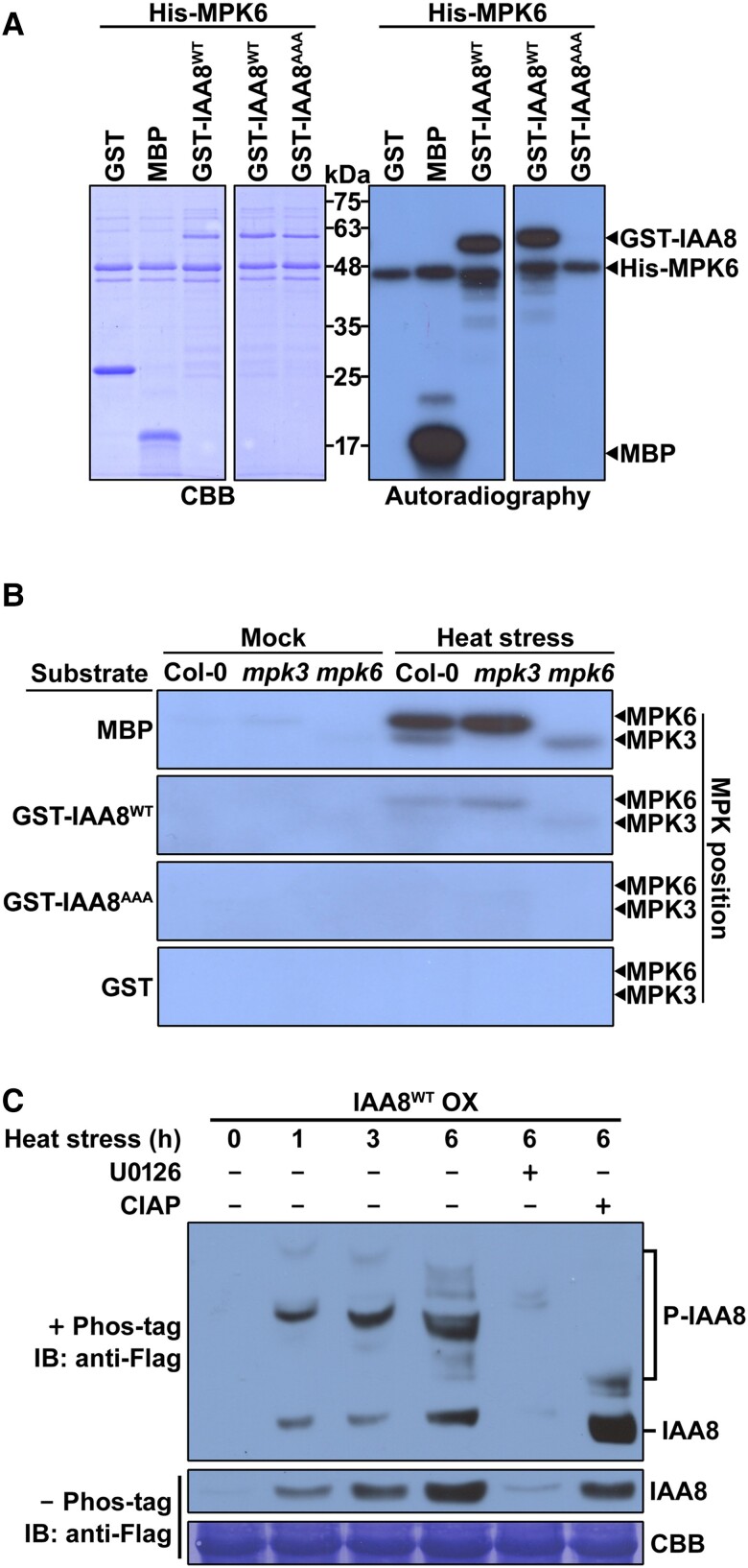
IAA8 is phosphorylated by MPKs in vitro and in vivo. **A)***In vitro* phosphorylation of GST-IAA8^WT^ and GST-IAA8^S74A/T77A/S135A^ (IAA8^AAA^) by recombinant His-MPK6. The recombinant proteins were incubated in a kinase buffer containing [γ-^32^P] ATP and resolved by 10% SDS-PAGE. Phosphorylated IAA8 and recombinant proteins were observed by autoradiography (right panel) and CBB staining (left panel), respectively. MBP and GST served as positive and negative control substrates, respectively. “−” and “+” indicate absence and presence, respectively. **B)***In-gel* kinase assay of MPK3 and MPK6 in response to heat stress. Total protein extracts from 3-week-old Col-0, *mpk3*, and *mpk6* plants subjected to heat (29 °C) for 10 min to activate MPK were resolved in MBP-, GST-IAA8^WT^-, GST-IAA8^AAA^-, or GST-embedded gels. MPK activities were detected with autoradiography, showing the expected locations of MPK3 and MPK6. **C)** Phos-tag mobility shift assay reveling in planta phosphorylation of IAA8 under heat stress conditions. IAA8^WT^ OX plants were treated with heat stress pulse with or without U0126. Total protein extracts from IAA8^WT^ OX plants were resolved in the gels embedded with (upper panel) or without (middle panel) phos-tag reagent after treatment with or without CIAP. Immunoblotting (IB) using anti-Flag antibodies was performed to observe IAA8 proteins. The CBB staining showed the Rubisco band as a loading control (lower panel).

Since IAA8 has been reported to be phosphorylated by MPK3 (Kim et al. 2022), we tested whether MPK4 and MPK6 can also phosphorylate IAA8. The GST-fused IAA8 and His-fused MPKs were used for the in vitro kinase assay. As a result, GST-IAA8 and myelin basic protein (MBP) (positive control) were phosphorylated by all used MPKs, but GST (negative control) was not (Fig. 2A and Supplementary Fig. S4), indicating that IAA8 is phosphorylated by three MPKs.

MPKs commonly phosphorylate the serine (Ser, S) and threonine (Thr, T) residues on their substrates, mostly at the S/T-P motifs. Five residues (S16, S63, S74, T77, and S135) of IAA8 were predicted as potential phosphorylation sites by MPKs (Supplementary Fig. S5). To identify the phosphorylation sites of IAA8 by MPKs, we performed mass spectrometry after the enrichment of phosphopeptides using TiO_2_ chromatography. Subsequently, seven phosphopeptides were isolated and analyzed (Supplementary Fig. S6 and Supplementary Table S1), revealing that three phosphorylation sites, S74, T77, and S135, were commonly found in five phosphopeptides: ETDFGLLS_74_PR, LGLPESQSPERETDFGLLS_74_PR, T_77_PDEKLLFPLLPSK, GSVRPGGGINMMLS_135_PK, and GFADTWDEFSGVKGSVRPGGGINMMLS_135_PK. To validate these three phosphorylation sites of IAA8, we performed an in vitro kinase assay after site-directed mutagenesis of three phosphorylation sites to alanine (Ala, A) (GST-IAA8^S74A/T77A/S135A^, referred to as GST-IAA8^AAA^). As a result, the phosphorylation of GST-IAA8^AAA^ by MPK6 nearly disappeared compared to that of GST-IAA8^WT^ (Fig. 2A), indicating that these residues are the phosphorylation sites of IAA8 by MPKs.

In order to ascertain whether plant MPKs can phosphorylate IAA8, we conducted an *in*-*gel* kinase assay with MBP (positive control), GST-IAA8^WT^, GST-IAA8^AAA^, and GST (negative control) as the embedded kinase substrates. Total proteins were extracted from 10-day-old Col-0, *mpk3*, and *mpk6* plants subjected to heat stress for 10 min. As a result, radiolabeled phosphorylated IAA8 bands were detected at the positions of MPK3 and MPK6 in the GST-IAA8^WT^-embedded gel (Fig. 2B). Expectedly, these phosphorylation patterns were observed in MBP-embedded gel but not in GST- and GST-IAA8^AAA^-embedded gels (Fig. 2B). Conclusively, these observations suggest that plant MPKs phosphorylate IAA8.

To further examine whether IAA8 is phosphorylated in planta, we performed a phos-tag mobility shift assay. Transgenic plants overexpressing wild-type *IAA8* (IAA8^WT^ OX) were generated and subjected to heat stress to activate MPKs in the absence and presence of U0126. In response to heat stress, IAA8 began to accumulate at 1 h, but this accumulation was diminished by co-treatment with U0126 (Fig. 2C, middle panel), indicating that IAA8 accumulation is induced by the MPK cascade. In the phos-tag gel, noticeably shifted bands (P-IAA8) were observed following heat stress, but these bands almost disappeared with the application of calf intestine alkaline phosphatase (CIAP) (Fig. 2C, upper panel), indicating that IAA8 is phosphorylated by heat stress and subsequently stabilized through phosphorylation in planta.

### The phospho-mimicking mutant of IAA8 causes defective flower development

Next, we explored the physiological relevance of IAA8 phosphorylation by further constructing transgenic plants overexpressing *IAA8^AAA^* (a nonphosphorylatable form) and *IAA8^DDD^* (a phosphor-mimicking form). After assessing the mRNA levels of IAA8 through RT-qPCR, three independent transgenic lines were selected for subsequent experiments. All transgenic plants exhibited comparable high transcript levels of *IAA8* (Supplementary Fig. S7A). However, IAA8 proteins in IAA8^WT^ OX and IAA8^AAA^ OX plants were not observed under mock conditions but were highly detected by the treatment with MG132 (Supplementary Fig. S7B). Intriguingly, IAA8 was highly abundant in IAA8^DDD^ OX plants, even in the mock conditions (Supplementary Fig. S7B), indicating that IAA8^DDD^ is more stable than IAA8^WT^ under normal conditions.

**Figure 3.**
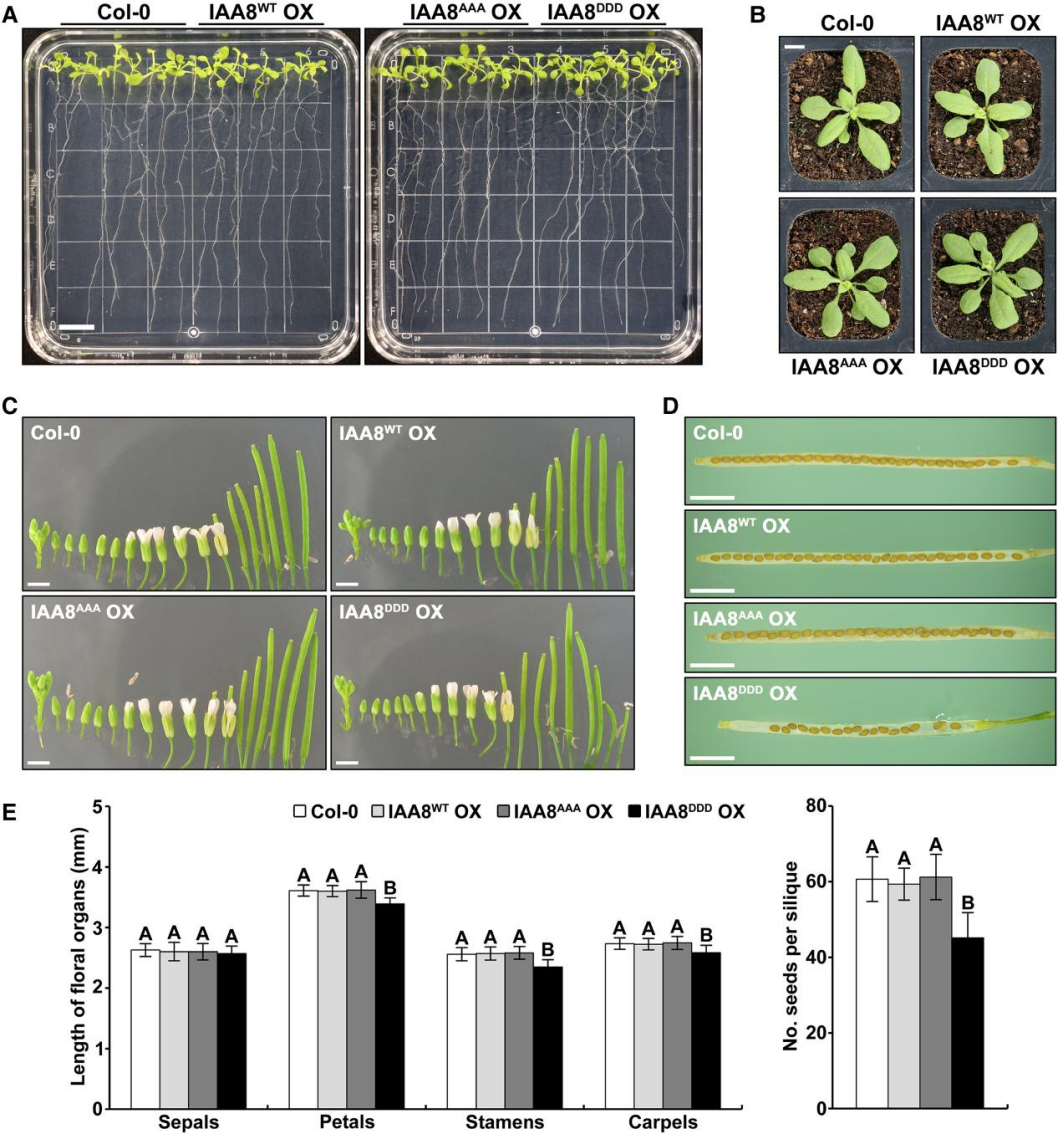
Transgenic plants overexpressing the IAA8 phospho-mimicking mutant show altered flower development. **A)** Root phenotypes of Col-0, IAA8^WT^ OX #2-1, IAA8^AAA^ OX #4-7, and IAA8^DDD^ OX #8-9 plants. Twelve-day-old plants grown vertically on MS plates were photographed. The scale bar represents 1 cm. **B)** Rosette leaves phenotypes of 4-week-old Col-0, IAA8^WT^ OX, IAA8^AAA^ OX, and IAA8^DDD^ OX plants grown in soil. The scale bar represents 1 cm. **C)** Floral phenotypes of Col-0, IAA8^WT^ OX, IAA8^AAA^ OX, and IAA8^DDD^ OX plants. The image displays flower buds progressing to siliques. The scale bars represent 1 mm. **D)** Numbers of seeds per mature silique in these transgenic plants. The scale bars represent 1 mm. **E)** Statistical analysis of the floral organ lengths and seed numbers in Col-0, IAA8^WT^ OX, IAA8^AAA^ OX, and IAA8^DDD^ OX plants. Floral organ lengths were measured at flower stage 14 of 35-day-old plants. Seed numbers were counted from the mature siliques. The bars indicate the mean ± SD (*n* = 50 flowers from two independent biological replicates). Different letters indicate significant differences between genotypes by one-way ANOVA followed with a Tukey test (*P* < 0.01).

Since IAA8 is unstable under normal conditions, the IAA8 loss-of-function mutant previously exhibited similar phenotypes to Col-0 (Overvoorde et al. 2005). In contrast, the IAA8 gain-of-function mutant was known to exhibit pleiotropic developmental phenotypes (Nagpal et al. 2005). Therefore, we investigated the developmental phenotypes of the transgenic plants. However, all transgenic plants did not show obvious developmental differences in roots and rosette leaves during the vegetative stage (Figs. 3, A and B and Supplementary Fig. S8). Interestingly, IAA8^DDD^ OX plants displayed obvious shortened floral organs compared to the other transgenic plants during the reproductive stage. The lengths of the petals, stamens, and carpels in the flowers of IAA8^DDD^ OX were significantly shorter than those in Col-0, IAA8^WT^ OX, and IAA8^AAA^ OX plants (Fig. 3, C and E, and Supplementary Fig. S9, A to D). Furthermore, short siliques and reduced seeds were also observed in IAA8^DDD^ OX plants (Figs. 3, D and E, and Supplementary Fig. S9E). These findings suggest that the phospho-mimicking mutant of IAA8 leads to defects in flower development, indicating a negative role of stabilized IAA8 in flower development. Since three independent transgenic lines of each genotype exhibited comparably high transcript levels and similar phenotypes (Supplementary Figs. S7 to S9), we chose IAA8^WT^ OX #2-1, IAA8^AAA^ OX #4-7, and IAA8^DDD^ OX #8-9 as representative lines, referred to hereafter as IAA8^WT^ OX, IAA8^AAA^ OX, and IAA8^DDD^ OX, respectively.

**Figure 4.**
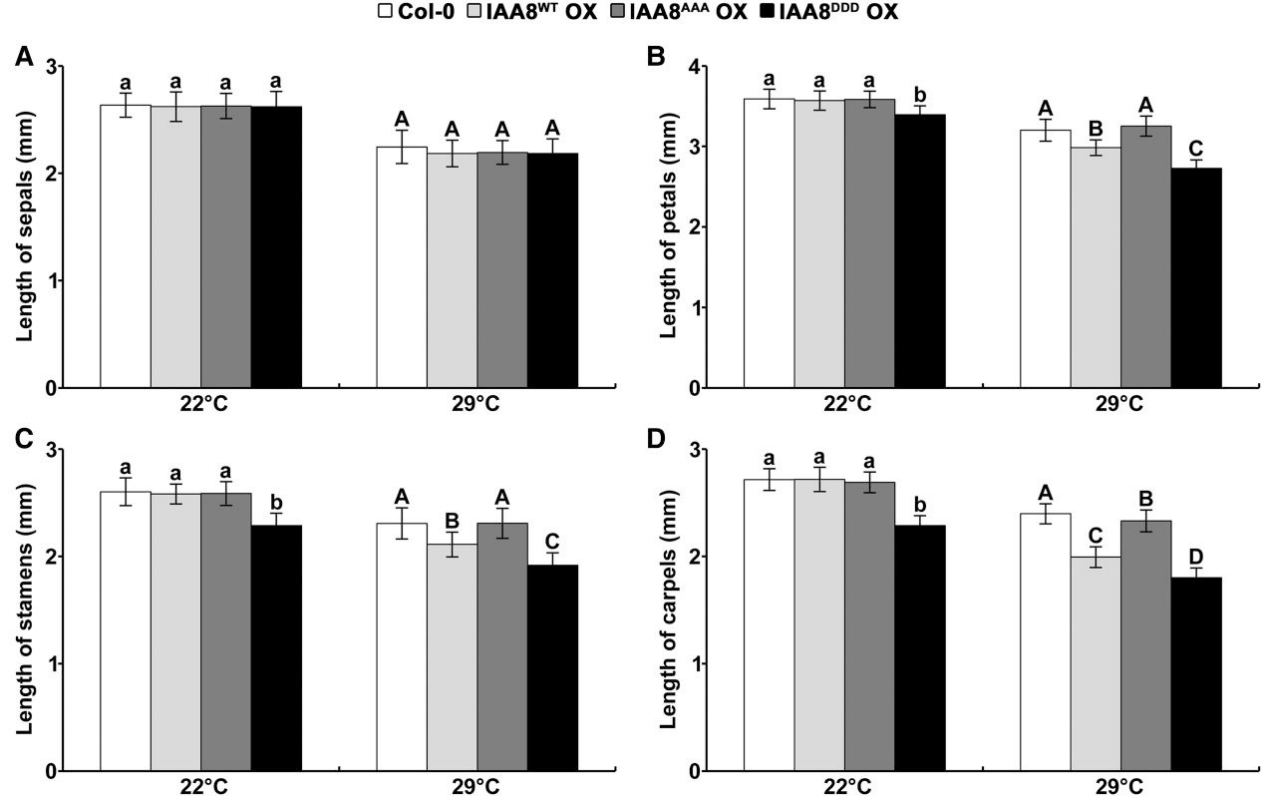
Phosphorylation of IAA8 mediates inhibition of floral organ development under heat stress. Length of sepals **(A)**, petals **(B)**, stamens **(C)**, and carpels **(D)** in IAA8 transgenic plants under normal (22°C) and heat stress (29°C) conditions. The organ lengths were measured at flower stage 14 of 35-day-old plants. The bars indicate the mean ± SD (*n* = 50 flowers from two independent biological replicates). Different letters indicate significant differences between genotypes by one-way ANOVA followed with a Tukey test (*P* < 0.01).

To identify the physiological function of IAA8 phosphorylation in response to heat stress, we measured the lengths of floral organs in IAA8 transgenic plants after the treatment with heat stress. Expectedly, the lengths of floral organs were significantly reduced in IAA8^DDD^ OX plants regardless of heat stress compared to in IAA8^WT^ and IAA8^AAA^ OX plants (Fig. 4). Interestingly, IAA8^WT^ OX plants exhibited shorter floral organs than IAA8^AAA^ OX plants under heat stress conditions (Fig. 4 and Supplementary Fig. S10), suggesting that MPK-mediated phosphorylation of IAA8 induces a heat-mediated defect in flower development. These results indicate that MPK-mediated phosphorylation of IAA8 negatively regulates flower development under heat stress conditions.

### Heat-responsive MPK increases the stability of IAA8 through phosphorylation

**Figure 5.**
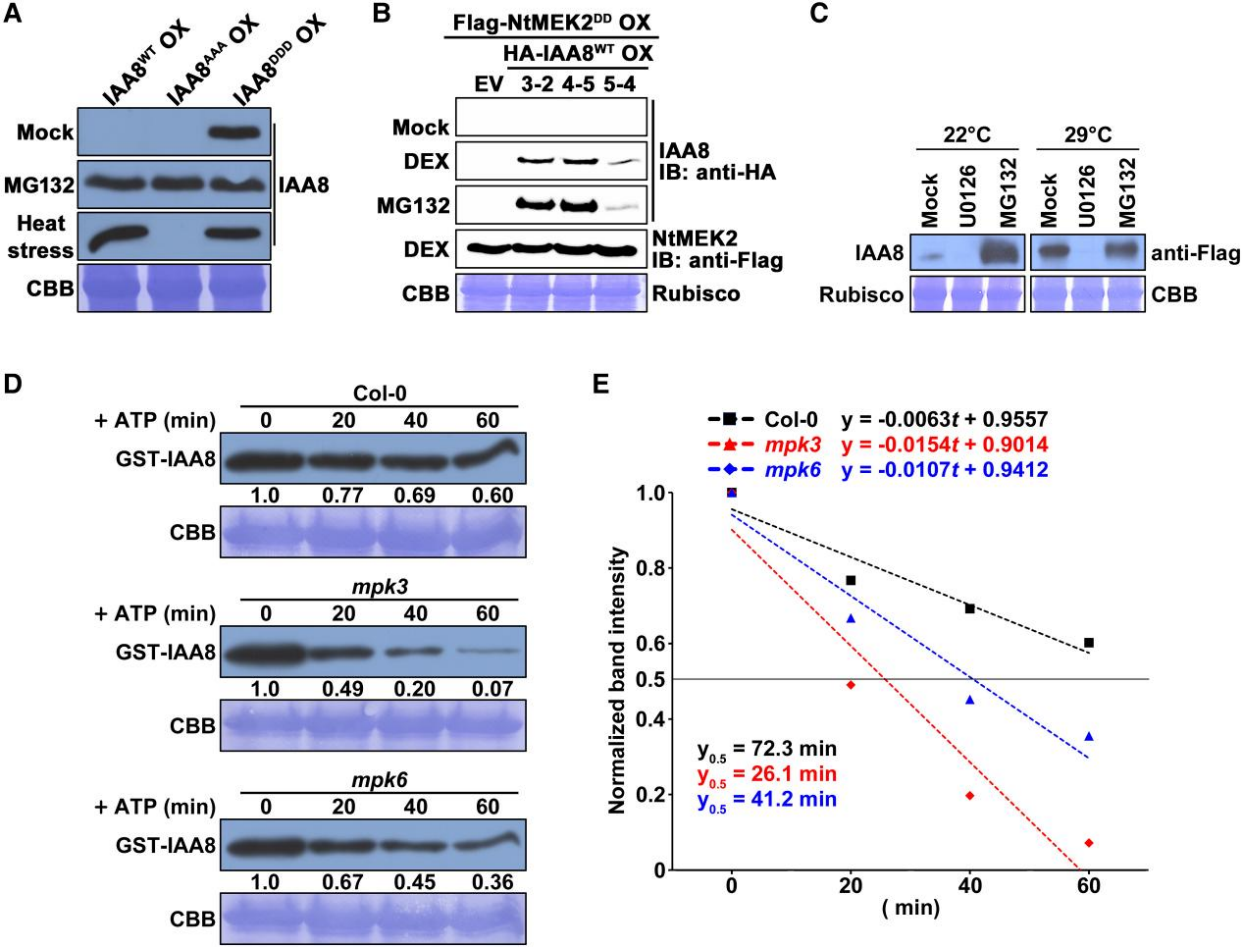
Heat stress induces the stabilization of IAA8 through MPK phosphorylation. **A)** IAA8 proteins in transgenic plants treated with mock, MG132, and heat stress was. Immunoblotting using anti-Flag antibodies was performed to observe IAA8 proteins. The representative CBB staining showed the Rubisco band as a loading control. **B)** Stabilization of IAA8 by the induction of constitutively active tobacco MEK2 (NtMEK2^DD^). Immunoblotting analyses were conducted in double transgenic plants expressing HA-tagged IAA8^WT^ and Flag-tagged NtMEK2^DD^ under treatment with mock, DEX, and MG132. Signals of immunoblots were detected by chemiluminescence using a ChemiDoc MP imaging system. The representative CBB staining showed the Rubisco band as a loading control. IB, immunoblotting. **C)** Inhibition of heat-induced stabilization of IAA8 by U0126. IAA8^WT^ OX plants treated with MG132 or U0126, were applied to heat stress, and immunoblot analysis was performed with anti-Flag antibodies to detect IAA8 proteins. **D)** Inhibition of IAA8 degradation by MPK3 and MPK6 in a cell-free protein degradation assay. Total protein extracts from 14-day-old Col, *mpk3*, and *mpk6* plants were incubated with GST-IAA8 and ATP. IAA8 proteins were detected using anti-GST antibodies. The CBB staining showed the Rubisco band as a loading control (lower panel). **E)** Linear regressions of the immunoblot band intensities **(D)** measured by ImageJ representing the degradation rates of GST-IAA8. The line and value of y_0.5_ indicate the half-life of GST-IAA8 proteins.

Most Aux/IAAs are well-known to be unstable due to their rapid turnover via the ubiquitin-proteasome pathway (Gray et al. 2001). However, the above result showed that IAA8^DDD^ protein was more stable than IAA8^WT^ and IAA8^AAA^ under normal conditions (Fig. 5A and Supplementary Fig. S7B). Furthermore, IAA8^WT^ was clearly stabilized under heat stress, whereas IAA8^AAA^ was not (Figs. 2, C and 5A, Supplementary Fig. S7), which suggests that MPK-mediated phosphorylation stabilize IAA8. To confirm this hypothesis, we constitutively expressed hemagglutinin (HA)-IAA8^WT^ under the control of the *35S* promoter in a dexamethasone (DEX)-inducible NtMEK2^DD^ OX plant background (Yang et al. 2001). These HA-IAA8^WT^/Flag-NtMEK2^DD^ OX double transgenic plants were treated with MG132 to inhibit IAA8 degradation or with DEX to induce the expression of NtMEK2^DD^ that activates MPKs in Arabidopsis. As expected, IAA8^WT^ protein was not detected under mock treatment but was highly detected upon MG132 treatment (Fig. 5B). Notably, IAA8^WT^ was also accumulated by DEX treatment (Fig. 5B), indicating that MPK-mediated phosphorylation induces the stabilization of the IAA8.

Since heat stress is known to activate MPK signaling (Evrard et al. 2013), we speculated that the heat-induced stabilization of IAA8 is mediated by MPK phosphorylation. To test this speculation, we examined the effects of U0126 on the heat-induced stabilization of IAA8. The heat-induced stabilization of IAA8 was abolished by U0126 treatment (Fig. 5C), suggesting that activated MPKs are essential for the heat-induced stabilization of IAA8.

Additionally, to confirm whether MPK3 and MPK6 regulate the stabilization of IAA8, we examined the degradation rates of recombinant GST-IAA8 proteins through cell-free protein degradation assays. As a result, we showed that GST-IAA8 was more rapidly degraded in the single *mpk3* and *mpk6* mutants compared to Col-0 (Fig. 5D). The half-life of GST-IAA8 proteins was 72.3 min in Col-0, whereas it was dropped to 26.1 min in *mpk3* and 41.2 min in *mpk6* (Fig. 5E), indicating that MPK3 and MPK6 redundantly regulate the heat-induced stabilization of IAA8. These findings suggest that the heat-induced stabilization of IAA8 by MPKs causes defects in flower development.

### MPK-mediated phosphorylation inhibits the polyubiquitination of IAA8

To understand the molecular mechanism of how IAA8 is stabilized by MPK phosphorylation, we initially analyzed the effects of MPK phosphorylation on the interactions between TIR1 and IAA8 variants using LCI assays. However, we could not observe any differences in their interactions regardless of auxin (Supplementary Fig. S11), suggesting that phosphorylation does not influence the interaction of TIR1 with IAA8.

**Figure 6.**
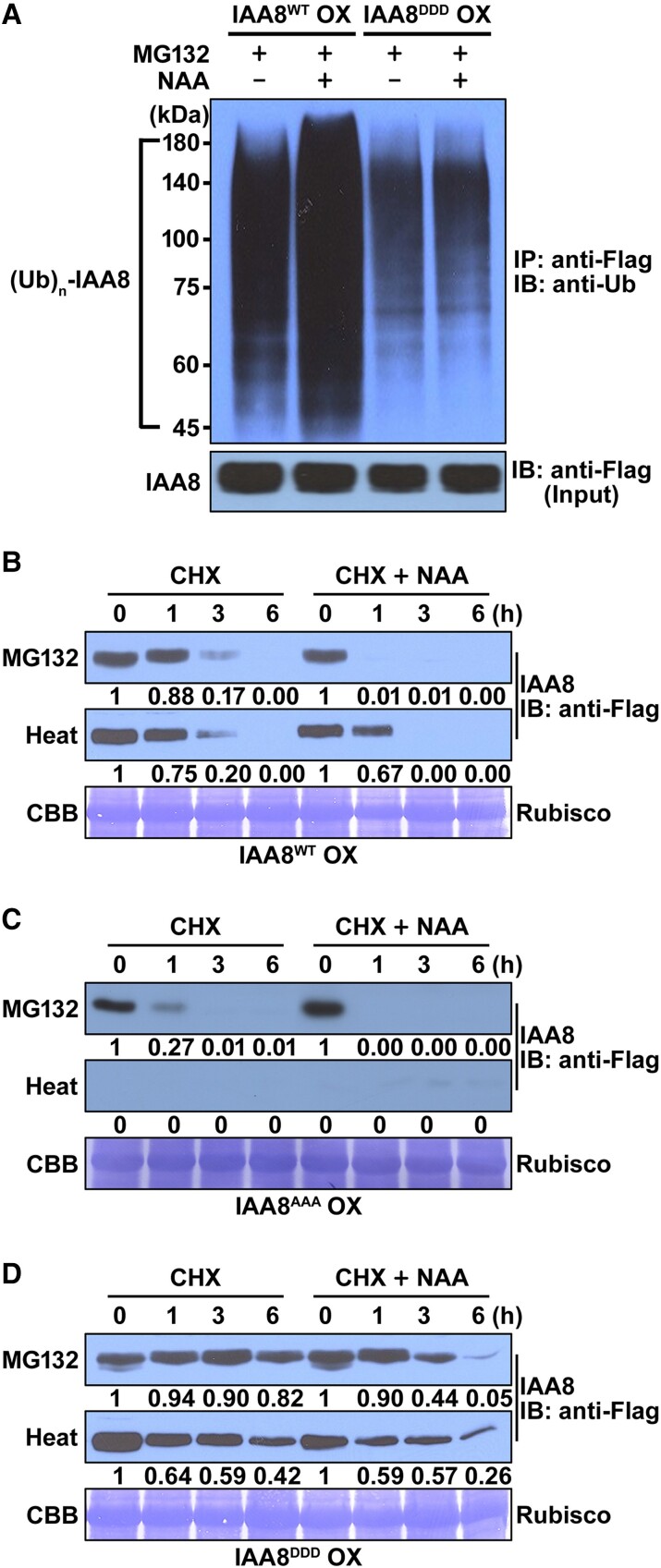
Phosphorylation of IAA8 inhibited its polyubiquitination. **A)** Auxin-induced polyubiquitination of IAA8^WT^ and IAA8^DDD^ in planta. Immunoblotting (IB) using antiubiquitin (anti-Ub) antibodies was performed to detect polyubiquitinated IAA8 after immunoprecipitation (IP) with anti-Flag antibodies from IAA8^WT^ OX and IAA8^DDD^ OX plants treated with 10 μM MG132 alone or in combination with 20 μM NAA (upper panel). A moderately high concentration of NAA (20 μM) was applied to amplify the difference in polyubiquitination. Immunoblotting (IB) using anti-Flag antibodies was performed to detect IAA8 proteins (lower panel, 10% input). “−” and “+” indicate absence and presence, respectively. **B–D)** Degradation rates of IAA8^WT^, IAA8^AAA^, and IAA8^DDD^ proteins in planta. Fourteen-day-old IAA8^WT^ OX **(B)**, IAA8^AAA^ OX **(C)**, and IAA8^DDD^ OX **(D)** plants, pretreated with 10 μM MG132 or pre-exposed to heat stress for 18 h, were then applied with or without 10 μM NAA in combination with 1 mm CHX for the indicated times. Total protein extracts from the transgenic plants were performed for immunoblotting with anti-Flag antibodies. The representative CBB staining showed the Rubisco band as a loading control.

Next, to examine whether the phosphorylation of IAA8 make an effect on its polyubiquitination by the ubiquitin-E3 ligase complex, we performed the in planta polyubiquitination of IAA8 in IAA8^WT^ OX and IAA8^DDD^ OX plants. As expected, the polyubiquitination of IAA8 was prominently detected in IAA8^WT^ OX plants, and the polyubiquitination levels of IAA8 were obviously increased upon NAA treatment (Fig. 6A, upper panel). However, the polyubiquitination levels of IAA8 were lower in the IAA8^DDD^ OX plants compared to in IAA8^WT^ OX plants regardless of NAA treatment (Fig. 6A, upper panel), indicating that the phosphorylation of IAA8 by MPK inhibits its polyubiquitination.

To further verify that phosphorylated IAA8 is more stable than its unphosphorylated form, we performed a protein turnover assay in IAA8^WT^ OX, IAA8^AAA^ OX, and IAA8^DDD^ OX plants. To prevent the degradation of IAA8 proteins by the 26S proteasome, these plants were pretreated with MG132 for 18 h and then applied with NAA in combination with cycloheximide (CHX) for 6 h. As a result, we found that IAA8^WT^ proteins were rapidly degraded within 3 h and even more quickly by NAA treatment (Fig. 6B). Interestingly, IAA8^DDD^ proteins were more stable than IAA8^WT^, but IAA8^AAA^ proteins were less stable irrespective of NAA treatment (Fig. 6, C and D). Moreover, similar patterns were observed when heat stress was applied to IAA8^WT^ OX and IAA8^DDD^ OX plants (Fig. 6, B to D). These results demonstrate that the phosphorylation of IAA8 by MPK inhibits its polyubiquitination.

### Expressions of auxin-responsive genes were inhibited by the phosphorylation of IAA8

In general, Aux/IAAs act as transcriptional repressors in auxin signaling. Therefore, it is expected that the stabilized IAA8 by phosphorylation may inhibit the auxin response. To test this possibility, we measured GUS activity in leaves of *DR5*::*GUS* transgenic plants co-expressing IAA8^WT^, IAA8^AAA^, and IAA8^DDD^. In the absence of NAA, we did not observe any significant difference in GUS activities among all transgenic plants (Supplementary Fig. S12). However, GUS activities in *DR5*::*GUS*, *DR5*::*GUS*/IAA8^WT^ OX, and *DR5*::*GUS*/IAA8^AAA^ OX plants were significantly increased upon treatment with NAA but not increased in *DR5*::*GUS*/IAA8^DDD^ OX plants. These results mean that the auxin response is inhibited by the phosphorylation of IAA8.

To examine whether the phosphorylation of IAA8 suppresses the expressions of auxin-responsive genes (such as *IAA2*, *IAA7*, *IAA19*, and *GH3.3*), we examined their expressions in IAA8^WT^ OX and IAA8^DDD^ OX plants using RT-qPCR analysis. Consistent with the auxin response, the expressions of auxin-responsive genes were highly reduced in IAA8^DDD^ OX plants compared to the IAA8^WT^ OX plants regardless of NAA (Supplementary Fig. S13). These results strongly support the notion that the auxin response is inhibited by the phosphorylation of IAA8 through the transcriptional down-regulation of auxin-responsive genes.

### IAA8^DDD^ OX plants show transcriptional perturbations of flower development-related genes

Since the gain-of-function mutant of IAA8 exhibited defective floral organs due to the reduction of JA biosynthesis (Wang et al. 2013), we suspected that the defective floral organs of IAA8^DDD^ OX plants under normal conditions could also be attributed to reduced JA levels. To test this possibility, we analyzed the transcript levels of JA biosynthesis-related genes, such as *DAD1*, *AOS*, *AOC4*, and *OPR3*, in flowers of IAA8 transgenic plants. However, the transcript levels of these genes were not changed (Supplementary Fig. S14A). Additionally, the JA levels were also similar in all transgenic plants (Supplementary Fig. S14B), indicating that the defective flower development in IAA8^DDD^ OX plants is not caused by the decrease of JA biosynthesis. These results suggest that the defective flower development in IAA8^DDD^ OX plants may have resulted from other pathways and genes involved in flower development.

**Figure 7.**
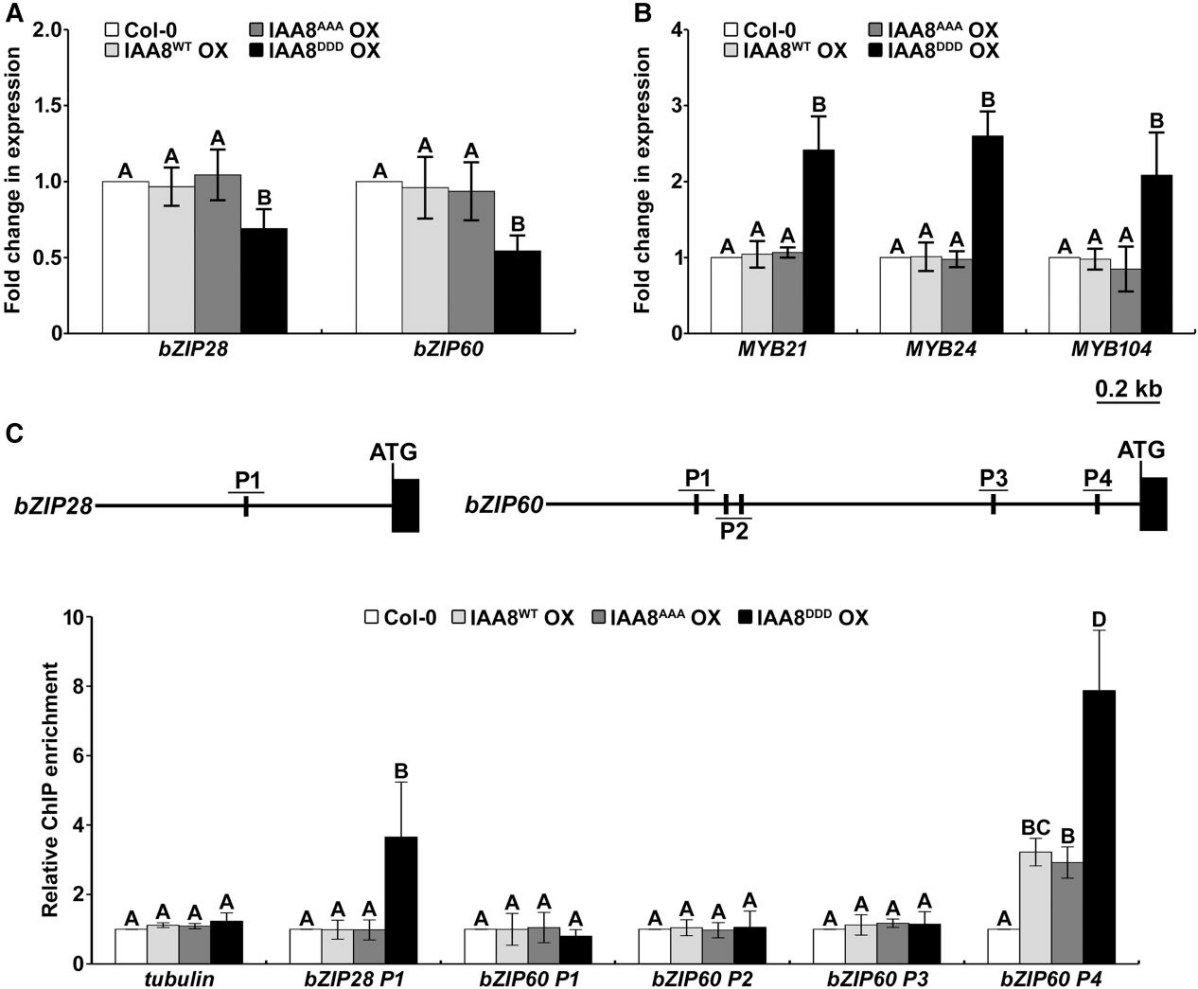
Perturbed transcript levels of flower development-related genes cause defects in flower development of IAA8^DDD^ OX plants under normal conditions. **A, C)** The transcript levels of *bZIPs***(A)** and *MYBs***(C)** genes were analyzed by RT-qPCR. Total RNAs were extracted from mature flowers of Col-0 and IAA8 transgenic plants (40-day-old) grown under normal conditions. RT-qPCR analyses were performed using gene-specific primers. The bars indicate the mean ± SD (*n* = 3). Different letters indicate significant differences between genotypes between genotypes by one-way ANOVA followed with a Tukey test (*P* < 0.01). **B)** IAA8 is directly bound to the promoters of *bZIP28* and *bZIP60*. Schematic diagrams of AuxREs, including TGTC in *bZIP28* promoter (P1) and *bZIP60* promoter (P1 to P4) regions are presented (upper). The 1.0 and 2.0 kb upstream sequence of the *bZIP28* and *bZIP60* promoters were shown, with the translational start sites (ATG) indicated at position +1, respectively. ChIP assays were conducted using anti-Flag antibodies with chromatin extracted from transgenic plants. Subsequently, qPCR was performed on the ChIP-DNA using primers specifically targeting the promoter regions of *bZIP28* and *bZIP60*. Tubulin used as negative control. The bars indicate the mean ± SD (*n* = 3). Different letters indicate significant differences between genotypes by one-way ANOVA followed with a Tukey test (*P* < 0.05).

Several previous studies have reported that different classes of TFs are involved in flower development. For instance, two membrane-associated TFs, *bZIP28* and *bZIP60*, play positive roles in flower development under heat stress (Zhang et al. 2017). Therefore, to elucidate whether their transcript levels are different in the flowers of Col-0 and IAA8 transgenic plants, we measured their transcript levels under normal conditions. As a result, we found that the transcript levels of *bZIP* genes were significantly lower in IAA8^DDD^ OX plants compared to those in Col-0, IAA8^WT^ OX, and IAA8^AAA^ OX plants (Fig. 7A) because only IAA8^DDD^ is stable under these conditions, indicating that stabilized IAA8 negatively regulates the transcription of these *bZIP* genes. Since typical auxin response elements (AuxREs) were found in the promoter regions of *bZIP28* (P1) and *bZIP60* (P1 to P4) genes, we examined the association of IAA8 with those elements by a chromatin immunoprecipitation (ChIP) assay. As a result, we revealed that IAA8 directly binds to AuxREs in the P1 region of the *bZIP28* promoter and the P4 region of the *bZIP60* promoter (Fig. 7B). These findings demonstrate that the stabilized IAA8 negatively regulates *bZIP* gene expressions through direct associations with promoters.

Meanwhile, optimal expressions of some *MYB* genes, such as *MYB21*, *MYB24*, and *MYB108,* have been exhibited to be required for flower development (Cheng et al. 2009; Mandaokar and Browse 2009). However, the expressions of *MYB* genes were significantly higher in IAA8^DDD^ OX plants compared to other transgenic plants (Fig. 7C). Overall, these findings suggest that defective flower development in IAA8^DDD^ OX plants is caused by the transcriptional perturbations of flower development genes.

## Discussion

Heat stress is predicted to be a disastrous environmental threat to agricultural productivity in the near future. Due to ongoing global warming, plants (including field crops) will be exposed to more serious heat stress, resulting in severe problems in crop yield. Actually, reproductive organs are known to be more sensitive to heat stress than vegetative organs (Barnabás et al. 2008; Begcy and Dresselhaus 2018; Chaturvedi et al. 2021). However, the underlying signaling pathway and molecular mechanism of how plants sense heat stress and consequently exhibit inhibited flower development remain to be elucidated. In this study, we demonstrate that phosphorylation of IAA8 by heat-responsive MPKs causes developmental defects in flowers due to transcriptional perturbations of genes regulating flower development. This study sheds light on the signaling pathway to explain how flower development deteriorates in response to environmental stresses.

### An MPK-IAA8 module triggers the heat-mediated suppression of auxin signaling in flower development

Previous studies have shown that elevated temperatures suppress the auxin response by decreasing auxin levels, which leads to defects in pollen development and filament elongation (Sakata et al. 2010). Actually, the floral defects caused by heat stress were restored by exogenous auxin treatment (Sakata et al. 2010), suggesting that heat stress down-regulates auxin biosynthesis during flower development. However, the molecular effects of heat stress on auxin signaling have not yet been extensively investigated. In this study, we showed that the heat-mediated inhibition of auxin signaling was rescued by treatment with U0126 (Supplementary Fig. S1), indicating that the suppression of auxin signaling by heat stress is mediated by MPK cascades.

In general, MPK cascades are widely involved in responses to environmental stresses through the phosphorylation of their substrate (Colcombet and Hirt 2008; Kim et al. 2017; Chen et al. 2021; Liu et al. 2022a). Interestingly, several Aux/IAAs have been reported as potential substrates of MPK3 (Kim et al. 2022). In this study, we showed that IAA8 is a bona fide substrate of heat-responsive MPKs through various assays (Fig. 2 and Supplementary Fig. S4). The Aux/IAA-mediated inhibition of auxin signaling under environmental stress conditions has been reported. For instance, IAA15 suppresses auxin signaling under drought conditions, inhibiting lateral root development (Kim et al. 2022). Similarly, IAA7 inhibits auxin signaling in response to salicylic acid (SA), thereby promoting pathogen resistance (Wang et al. 2007). The inhibition of auxin signaling has also been achieved by transcriptional induction of Aux/IAA genes under drought conditions (Shani et al. 2017; Salehin et al. 2019). Therefore, this study contributes to our understanding of how the post-translational modifications (PTMs) of Aux/IAAs suppress auxin signaling to achieve developmental plasticity under various environmental stresses. To further clarify the mechanism by which Aux/IAAs are dedicated to particular environmental stresses, more specific studies on various Aux/IAAs should be conducted in the future.

### Phosphorylated IAA8 is stabilized by the inhibition of its polyubiquitination

Most Aux/IAAs undergo rapid polyubiquitination by the SCF^TIR1/AFB^ complex and are quickly degraded by the 26S proteasome (Kepinski and Leyser 2002; Dharmasiri et al. 2005; Calderón Villalobos et al. 2012). Given this characteristic, the regulation of their stabilities is considered essential for the physiological functions of Aux/IAAs under environmental stresses. In this study, we discovered that the stabilization of IAA8 by heat stress was abolished by U0126 treatment (Figs. 2C and 5C), indicating that the phosphorylation by heat-responsive MPKs directly contributes to the stabilization of IAA8. Similar to IAA8, it has been reported that the stabilities of several Aux/IAAs are regulated by PTMs of Aux/IAAs (Cui et al. 2024). The stabilities of noncanonical IAA32/IAA34 and IAA33 were also induced by the phosphorylation of TMK1 and MPK14, respectively (Cao et al. 2019; Lv et al. 2020). Furthermore, the stability of IAA17 was increased by both sumoylation and nitrosylation (Jing et al. 2023; Zhang et al. 2023). The IAA15 stability is induced by the MPK3/MPK6 phosphorylation in response to drought (Kim et al. 2022). Therefore, our results provide another aspect of evidence that the stabilization of Aux/IAAs is regulated by MPK phosphorylation in auxin signaling.

Furthermore, we found that MPK phosphorylation stabilizes IAA8 by inhibiting its polyubiquitination rather than affecting its interaction with TIR1 (Fig. 6A and Supplementary Fig. S11), suggesting that the interplay between phosphorylation and ubiquitination regulates the stability of IAA8. Numerous studies have reported the relationships between phosphorylation and ubiquitination in animals and plants (Lin et al. 2006; Ding et al. 2015; Garvin et al. 2019; Zhang and Zeng 2020). However, the precise mechanism by which phosphorylation inhibits polyubiquitination has not been elucidated. Previously, we reported that although the predicted phosphorylation sites (SP and TP) of several canonical IAA proteins by MPK3 were not conserved, these sites were located near the potential ubiquitination sites (Kim et al. 2022). Given that the putative ubiquitination site of IAA8, lysine-107 (Lys-107), is located in proximity to three phosphorylation sites (S74, T77, and S135) but far from the SCF^TIR1/AFB^ docking motif (Supplementary Fig. S5), we propose that the phosphate groups at these phosphorylation sites of IAA8 may prevent ubiquitination, possibly via conformational changes of IAA8. To understand the effects of phosphorylation on the structure of IAA8, we analyzed 3D structures of IAA8^WT^, phosphorylated IAA8^WT^ (P-IAA8^WT^), and IAA8^DDD^ through computer modeling. The structural analysis suggested that P-IAA8^WT^ and IAA8^DDD^ exhibit a different conformation from IAA8^WT^ (Supplementary Fig. S15), indicating that phosphorylation or phospho-mimicking mutation at positions of S74, T77, and S135 increases the overall negative charge of IAA8, directly causing its conformational changes. Otherwise, it is also possible that phosphorylation and ubiquitination compete for the same sites because Ser and Thr residues, in addition to Lys, can also be ubiquitinated in Aux/IAA proteins (Gilkerson et al. 2015). Therefore, identifying the specific ubiquitination sites would help elucidate how phosphorylation inhibits the polyubiquitination of IAA8.

### The MPK-IAA8 module causes defective flower development, presumably by the perturbed expressions of *bZIP* and *MYB* genes

Our study showed that the IAA8^DDD^ OX plant exhibits defects in flower development (Figs. 3 and 4). Defective flower development has been reported to be caused by reduced JA levels in both the IAA8 gain-of-function mutant and the ARF6/ARF8 loss-of-function mutant (Nagpal et al. 2005; Wang et al. 2013). Nevertheless, we observed no reduction of JA level in IAA8^DDD^ OX plant (Supplementary Fig. S14). Instead, we found that the expressions of *bZIPs* and *MYBs* genes were significantly reduced and induced in IAA8^DDD^ OX plants, respectively (Fig. 7, A and C), suggesting that the defective flower development in IAA8^DDD^ OX plants might be mainly attributed to perturbed expressions of *bZIP* and *MYB* genes. To clarify this finding, a detailed study elucidating the genetic interaction of IAA8 with *bZIPs* or *MYBs* in flower development needs to be conducted in the near future.

These results prompt inquiries into how phosphorylated IAA8 regulates the expression of *bZIP* and *MYB* genes. Since Aux/IAAs basically function as transcriptional repressors, it is possible that IAA8 directly down-regulates *bZIP* genes (Fig. 7B) and unknown transcriptional repressors that inhibit the transcription of *MYB* genes. Therefore, further study would be required to identify the downstream target gene of IAA8 potentially encoding these unknown repressors.

### Distinct phenotypes of plants expressing phospho-mimicking mutant and gain-of-function mutant of IAA8

Most Aux/IAA gain-of-function mutants have been reported to show pleiotropic auxin-deficient phenotypes (Reed 2001). Likewise, the IAA8 gain-of-function mutant has shown various developmental phenotypes (Wang et al. 2013). However, IAA8^DDD^ OX plants only exhibit a defective flower phenotype due to specifically perturbed expressions of *bZIP* and *MYB* genes (Figs. 3, 4, and 7). These results imply that the MPK-IAA8 module participates explicitly in the transcriptional regulation of flower development genes. These results also raise a question of why IAA8 gain-of-function mutant and IAA8^DDD^ OX plants exhibit different phenotypes. One possible explanation is that the IAA8 gain-of-function mutant was more abundantly accumulated than the IAA8^DDD^ mutant due to its substantially increased stability. When the degradation rates of recombinant GST-IAA8^DDD^ and GST-IAA8^P170L^ (a gain-of-function mutant of IAA8) were measured using cell-free protein degradation assays, we observed that the GST-IAA8^P170L^ protein is more stable than the GST-IAA8^DDD^ protein (Supplementary Fig. S16). A similar phenomenon has also been observed in the case of IAA15 (Kim et al. 2022). Therefore, the pleiotropic phenotypes of IAA8 gain-of-function mutants could be a consequence of the potentially excessive accumulation of IAA8, which resulted from the almost complete disruption of its interaction with TIR1 (Wang et al. 2013). In contrast, IAA8^DDD^ OX plants only showed the defective flower phenotype because IAA8 is moderately accumulated by moderately inhibited polyubiquitination (Fig. 6A and Supplementary Fig. S16).

**Figure 8.**
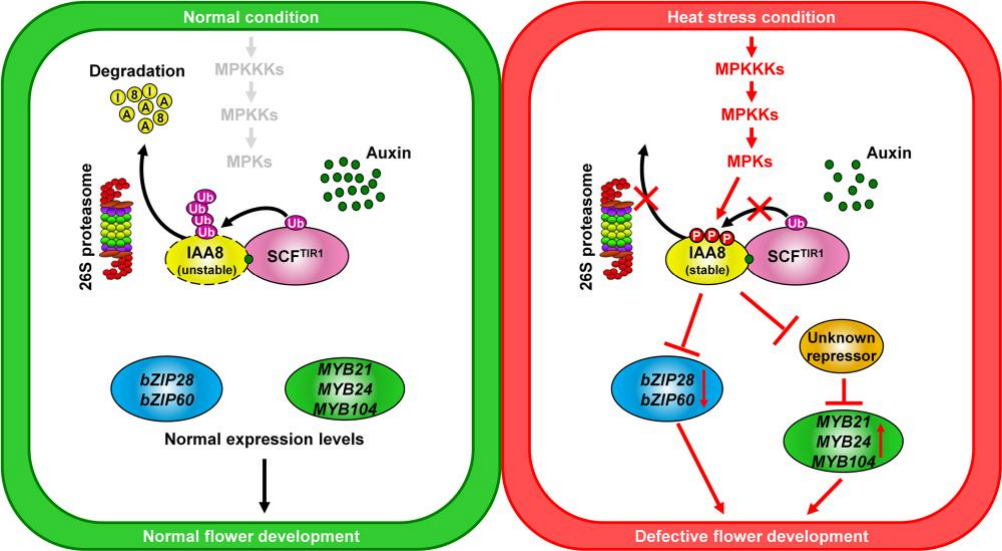
Proposed model explaining the inhibition of flower development by an MPK-IAA8 module under heat stress conditions. Under normal conditions (left panel), IAA8 is rapidly degraded through the ubiquitin-mediated 26S proteasome pathway, allowing for optimal expressions of *bZIP* and *MYB* genes and normal flower development. However, under heat stress conditions (right panel), IAA8 is stabilized by the phosphorylation of heat-responsive MPKs. This stabilized IAA8 inhibits the expressions of *bZIP28* and *bZIP60*. Conversely, IAA8 leads to a transcriptional increase of *MYB* genes, probably through the transcriptional suppression of unknown repressors, which may consequently cause defects in flower development. Solid lines ending with arrowheads represent activation, while those ending with perpendicular lines indicate inhibition. The crossed bars (X) indicate the blocking of the pointed signaling events.

In conclusion, we propose a model explaining how the phosphorylation of IAA8 inhibits flower development under heat stress (Fig. 8). Under normal conditions, IAA8 undergoes polyubiquitination by SCF^TIR1/AFB^ ubiquitin-E3 ligase complex, which leads to rapid degradation by the 26S proteasome. This process ensures normal flower development through the normal expressions of *bZIP* and *MYB* genes. However, under heat stress conditions, IAA8 is stabilized by phosphorylation of heat-responsive MPKs, resulting in the transcriptional inhibition of flower development-related *bZIP* genes. Simultaneously, the stabilized IAA8 may induce the expression of *MYB* genes through the transcriptional suppression of an unknown transcriptional repressor. This study provides a molecular mechanism for understanding how heat stress inhibits flower development, which could be applied to the development of heat-tolerant crops through genome editing of Aux/IAA genes. This model would be helpful in addressing crop productivity issues caused by climate change.

## Materials and methods

### Plant materials and growth conditions

In this investigation, Arabidopsis (*A. thaliana*) ecotype Columbia (Col-0) and *N. benthamiana* plants served as the experimental subjects. All genetic variations and genetically modified plants originated from the Col-0 ecotype. The previously documented mutants (*mpk3*, SALK_151594; *mpk6*, SALK_127507) and transgenic plants (*DR5::GUS OX* and NtMEK2^DD^ OX) were utilized in this study (Ulmasov et al. 1997; Liu and Zhang 2004; Ren et al. 2008). Seeds were sterilized and placed on agar plates containing 1/2 Murashige-Skoog (MS) salts, vitamins, and sucrose (20 g/L). Following a 3-day incubation at 4°C in darkness, plates were transferred to a growth chamber maintained at 22 °C, with a 16 h light/8 h dark cycle and a light intensity of approximately 120 μmol m^−2 ^s^−1^. Twelve- to 14-day-old seedlings were then transplanted into soil and kept under the same light and temperature conditions. To assess the impact of heat stress on flower growth and development, plants were moved to growth chambers set at 29°C, with a 16 h light/8 h dark cycle, for the specified duration.

### Expression and purification of recombinant proteins

Full-length IAA8 and MPKs were amplified via PCR using gene-specific primers obtained from an Arabidopsis cDNA library (Supplementary Table S2). The PCR products were cloned into T-blunt vectors (Solgent, Korea) and verified through sequencing. To construct GST-IAA8, inserts were excised with *Bam*HI and *Xba*I to insert into pGEX-4T-1 vectors (Amersham Biosciences, USA). Site-directed mutagenesis was utilized to create GST-IAA8^AAA^ and GST-IAA8^DDD^ variants using specific primers (Supplementary Table S2), with the mutated sequence confirmed by sequencing. The GST fusion constructs were introduced into the BL21 (DE3) strain of *E. coli*, and the resulting GST fusion proteins were expressed and purified using glutathione Sepharose-4B beads (Sigma-Aldrich, USA). For His-MPKs, inserts were excised with *Bam*HI and *Sal*I and inserted into pQE30 vectors (Qiagen, Germany). The resulting His-fusion constructs were transformed into *E. coli* (M15) strains and His-fusion proteins were expressed and purified using Ni-NTA agarose beads (Qiagen).

### In vitro pull-down assays

Following the protocol of Kim et al. (2017), a pull-down assay was conducted. A mixture of 2 μg of purified GST-IAA8 and GST combined with 10 μL of glutathione-Sepharose-4B beads in pull-down buffer (50 mm Tris-HCl, pH 7.5, 200 mm NaCl, 0.1 mm EDTA, 0.5 mm DTT, 1% (v/v) Triton X-100, and a protease inhibitor cocktail) were incubated for 3 h at 4°C. Subsequently, purified His-MPK3, -MPK4, or -MPK6 (at 5 μg) was introduced and incubated overnight at 4°C. The solution with beads was centrifuged, collected, and subjected to four washes with a pull-down buffer. The elution of proteins was accomplished by boiling in a loading buffer, followed by separation using 10% SDS-PAGE, transferred onto a PVDF membrane (Bio-Rad, USA), and visualized via immunoblotting using anti-His antibodies (Abcam Inc., UK).

### Yeast two-hybrid analysis

Full-length IAA8^WT^ was amplified using gene-specific primers (Supplementary Table S2) and subsequently cloned into pGAD424 (AD, Clontech), which harbors the *Leu2* selection marker. Similarly, MPK3, MPK4, and MPK6 were amplified with gene-specific primers (Supplementary Table S2) and cloned into pAS2-1 (BD, Clontech), carrying the selection marker *Trp1*. Following this, AD and BD plasmids were co-transformed into the yeast strain pJ69-4A (James et al. 1996) and plated onto SD/-Leu/-Trp (-L/W) medium to facilitate the selection of the introduced plasmids. Potential interactions between bait and prey were assessed by measuring the activation levels of the reporter genes, *HIS3* or *LacZ* through cellular growth and *β*-galactosidase activity assays, respectively.

### LCI assays

To investigate the in planta interactions between IAA8 and MPKs, complete sequences of IAA8 along with MPK3, MPK4, or MPK6 were cloned into pCAMBIA1300-NLuc and -CLuc vectors, resulting in the generation of *IAA8-NLuc* and *CLuc-MPKs* constructs, respectively. Similarly, to explore the in planta interaction between IAA8 and TIR1, *IAA8^WT^, IAA8^AAA^,* or *IAA8^DDD^*, along with TIR1, were cloned to pCAMBIA1300-NLuc and -CLuc vectors, leading to the generation of *IAA8^WT^-, IAA8^AAA^*-, or *IAA8^DDD^-NLuc* and *CLuc-TIR1* constructs, respectively. Each constructed plasmid was transformed into *Agrobacterium* strain GV3101 (pMP90). The *Agrobacterium* cells containing the various constructs were then infiltrated into the leaves of *N. benthamiana* plants, which were subsequently covered with plastic bags and incubated for 3 d at 25°C. Following this incubation period, the infiltrated leaves were sprayed with 100 μm D-luciferin and kept in the dark for 4 h to eliminate any background fluorescence. Finally, LUC activity levels were recorded using a low-light EMCCD apparatus (AndoriXon; Andor Inc., UK).

### In vitro and in-gel kinase assays

To catalyze the phosphorylation of GST (1 μg), MBP (0.5 μg), and GST-IAA8 variants (2 μg), His-MPKs were utilized in a kinase buffer (25 mm Tris-HCl (pH 7.5), 1 mm DTT, 20 mm MgCl_2_, 2 mm MnCl_2_, and 1 μCi of [γ-^32^P] ATP) and reacted for 30 min at 30°C. The reaction was stopped by adding 4× SDS sample buffer and boiling for 5 min. The reaction products were then separated using 10% SDS-PAGE, visualized through autoradiography, and Coomassie Brilliant Blue (CBB) staining.

For in-gel kinase assays, total protein was extracted from 2- to 3-week-old Col-0, *mpk3*, and *mpk6* plants subjected to heat stress (29°C) for 10 min. The total proteins (30 μg) were incubated at 60°C for 10 min and resolved on a 10% SDS-PAGE gel containing MBP (0.1 μg/μL), purified GST-IAA8 variants (0.7 μg/μL), or purified GST (0.7 μg/μL) as substrates. The gel was washed thrice with washing buffer (25 mm Tris-HCl, pH 7.5, 0.5 mm DTT, 0.1 mm Na_3_VO_4_, 5 mm NaF, 0.5 mg/mL bovine serum albumin, and 0.1% (v/v) Triton X-100) to remove SDS. Protein renaturation was achieved by overnight incubation of the gel in renaturing buffer (25 mm Tris-HCl, pH 7.5, 1 mm DTT, 0.1 mm Na_3_VO_4_, and 5 mm NaF) at 4°C. Following this, the gel was equilibrated in reaction buffer (25 mm Tris-HCl, pH 7.5, 2 mm EGTA, 12 mm MgCl_2_, 1 mm DTT, and 0.1 mm Na_3_VO_4_) at 30°C for 30 min before initiating the kinase reaction with 0.5 μm ATP and 50 μCi [γ-^32^P] ATP for 1.5 h at 30 °C. The reaction was stopped with a solution (5% (w/v) trichloroacetic acid and 1% (w/v) disodium pyrophosphate) and washed five times at 25 °C for 5 h. Subsequently, the gels were dried on 3 m paper, and phosphorylated proteins were recorded through autoradiography.

### Phos-tag mobility shift assays

Total proteins (80 μg) from 10-day-old IAA8^WT^ OX plants exposed to heat stress at 29°C for 0, 1, 3, and 6 h, with or without U0126, were separated on 10% SDS-PAGE gels containing 100 μM Phos-tag (NARD Institute, Japan) and 200 μM ZnCl_2_. After electrophoresis, the gel was rinsed twice with transfer buffer (added with 1 mm EDTA) for 10 min, and then proteins were transferred onto a PVDF membrane. IAA8 proteins were detected using anti-Flag antibodies (Sigma-Aldrich, USA).

### Generation of transgenic plants

For constructing IAA8^WT^ OX, IAA8^AAA^ OX, and IAA8^DDD^ OX plants, gene-specific sequences for IAA8^WT^, IAA8^AAA^, and IAA8^DDD^ were inserted into the pBlueScript II KS (+) vectors with a *3XFLAG* at *Bam*HI and *Xba*I sites (Stratagene, USA). These constructs of *3XFLAG*-*IAA8^WT^*, *3XFLAG*-*IAA8^AAA^*, and *3XFLAG*-*IAA8^DDD^* were cloned into pCAMBIA1300 vectors (Abcam) under the *35S* promoter. *Agrobacterium* strain GV3101 (pMP90) was used to introduce these constructs into Col-0 or *DR5::GUS* OX plants, and transformed plants were selected on MS media with hygromycin. In subsequent experiments, homozygous transgenic plants exhibiting enhanced IAA8 transcript and protein levels were used.

For Flag-NtMEK2^DD^/HA-IAA8^WT^ OX plants, the IAA8^WT^ gene was placed into a pMLBart vector with the HA epitope under the *35S* promoter. This construct was introduced into Flag-NtMEK2^DD^ OX plants, and the transformed plants were selected on MS medium containing phosphinothricin. Homozygous transgenic plants with increased IAA8 expression were used.

### Extraction and immunoblot analysis of Arabidopsis proteins

Total plant proteins were extracted using extraction buffer (50 mm Tris-HCl at pH 7.5, 5 mm EDTA, 5 mm EGTA, 1 mm Na_3_VO_4_, 25 mm NaF, 50 mm glycerophosphate, 2 mm DTT, 2 mm PMSF, 5% glycerol, 1% Triton X-100, and a protease inhibitor cocktail) and separated via SDS-PAGE. The visualization of the IAA8 protein was achieved through immunoblotting with anti-Flag antibodies, using X-ray film (AGFA, Belgium). In the case of Fig. 5B, signals of immunoblotting were detected by a ChemiDoc MP imaging system (Bio-Rad).

### In vivo turnover and ubiquitination assays

Fourteen-day-old IAA8^WT^ OX, IAA8^AAA^ OX, and IAA8^DDD^ OX plants were pretreated with 10 μM MG132 or subjected to heat stress for 18 h, washed with distilled water, and then exposed to 10 μM NAA in the presence of 1 mm CHX for 6 h. These plants were used to measure the in vivo degradation of IAA8 proteins. Following respective treatments, total proteins were extracted from the plants, separated by SDS-PAGE, and analyzed through immunoblotting using anti-Flag antibodies. To assess ubiquitination, total proteins were extracted from 10-day-old IAA8^WT^ OX and IAA8^DDD^ OX plans treated with or without 10 μM NAA in the presence of 10 μM MG132 for 24 h. These proteins were then immunoprecipitated using anti-Flag antibody-conjugated agarose beads (Sigma-Aldrich) at 4°C for 4 h. The beads were centrifuged, washed five times with extraction buffer, and eluted in sample buffer. The proteins were resolved on SDS-PAGE and analyzed by immunoblotting with antiubiquitin antibodies (Santa Cruz Biotechnology, USA).

### Cell-free protein degradation assay

Total cellular proteins were obtained from 14-day-old Col-0, *mpk3*, and *mpk6* plants to conduct a cell-free protein degradation analysis of IAA8. Equal amounts of total proteins were incubated with recombinant GST-IAA8 protein in degradation assay buffer (50 mm Tris-HCl at pH 8.0, 500 mm sucrose, 1 mm MgCl_2_, 10 mm EDTA at pH 8.0, and 5 mm DTT) at 25°C for various durations. Visualization and analysis of the IAA8 protein were performed through immunoblotting with anti-GST antibodies (Abm, USA).

### RT-qPCR analysis

Total RNA from plants was isolated using an RNA purification kit (Macherey-Nagel, Germany). Then, 5 μg of total RNA underwent reverse transcription using the SuperScript II RNase-Reverse Transcriptase assay (Invitrogen, USA). The RT-qPCR analysis was conducted on a CFX384 Real-Time System (Bio-Rad, USA) using iQ SYBR Green Supermix (Bio-Rad) and a unique set of primers for each gene (listed in Supplementary Table S3).

### ChIP assay

For the ChIP assay, 500 mg plant material from Col-0, IAA8^WT^ OX, IAA8^AAA^ OX, and IAA8^DDD^ OX was collected and fixed in sterilized H_2_O containing 1% formaldehyde. ChIP assays were conducted utilizing anti-Flag antibodies (dilution 1:3000) following established protocols (Bastow et al. 2004). qPCR analyses were carried out using the CFX384 Real-Time System with iQ SYBR Green Supermix and gene-specific primers (refer to Supplementary Table S3).

### Histochemical GUS assays

GUS assays were performed following the method described by Jefferson et al. (1987), with slight modifications. To examine GUS expression in flowers under heat stress, inflorescences of *DR5*::*GUS* plants were exposed to heat stress (29°C) with or without U0126. They were then incubated in a solution containing 2 mm 5-bromo-4-chloro-3-indolyl-β-D-glucuronic acid (X-gluc) in 50 mm phosphate buffer (pH 7.0), supplemented with 0.5 mm K_3_Fe(CN)_6_ and 0.5 mm K_4_Fe(CN)_6_ for 6 h at 37°C. Afterward, the tissue was rinsed with 50 mm phosphate buffer, fixed, and cleared overnight in ethanol (100%) and acetic acid (9:1, v/v) mixture at room temperature. The samples were imaged using a Nikon SMZ1000 stereoscopic microscope equipped with an OLYMPUS C-5050 ZOOM digital camera.

To explore IAA8-mediated GUS expression, levees of *DR5*::*GUS*, *DR5*::*GUS*/IAA8^WT^ OX, *DR5*::*GUS*/IAA8^AAA^ OX, and *DR5*::*GUS*/IAA8^DDD^ OX plants were treated with or without 10 µM NAA and then processed as described above.

### Mass spectrometric analysis of phosphopeptides using TiO_2_ microcolumns

GST-IAA8, treated with His-MPK6 in vitro kinase assays, were separated by SDS-PAGE, and the IAA8 protein bands were excised and in-gel digested with modified trypsin (Promega, USA) (Lee et al. 2004). The digested peptides were dissolved in a loading buffer (80% (v/v) acetonitrile and 5% (w/v) trifluoroacetic acid) and passed through a TiO_2_ microcolumn. The phosphopeptides eluted with NH_4_OH (pH 10.5) were applied to a Poros Oligo R3 column (Applied Biosystems, USA) and eluted using a 2,5-dihydroxybenzoic acid (DHB; Fluka, USA) solution (containing 20 mg/mL DHB, 50% (v/v) acetonitrile, 0.1% trifluoroacetic acid, and 1% (v/v) orthophosphoric acid). MALDI-MS analysis was performed on a Voyager-DE STR mass spectrometer (PerSeptive Biosystems, Inc., USA) set to the reflectron/delayed extraction mode. Mass spectra were analyzed using MoverZ software to identify monoisotopic peptide masses.

### The 3D structures of IAA8 and structural comparisons

The computational molecular modeling of IAA8^WT^ was conducted using the freely available *RoseTTAFold* webserver on Robetta (https://robetta.bakerlab.org/). The protein sequence of IAA8^WT^ was submitted as an input into the Robetta, which utilized a comprehensive protein structure database to generate five accurate PDB-format models via a folding method. The first model, with a confidence score of 0.40, was chosen as the potential 3D structure of IAA8^WT^. This structure was further refined using the *Steepest Descent* algorithm in *Discovery Studio v23* (*DS*) (BIOvIA Discovery Studio 2015). Phosphorylated IAA8^WT^ (P-IAA8^WT^) and phosphor-mimicking IAA8 (IAA8^DDD^) were prepared and minimized using the *Build and Edit Protein and Minimization* module in *DS* (Singh et al. 2022). For structural comparisons, the Align and Superimpose tool in *DS* was used.

### Statistical analysis

Statistical analyses were performed using Prism Graphpad software. The Student's *t*-test was used for comparisons between two groups (* for *P* < 0.05; ** for *P* < 0.01; *** for *P* < 0.001). The number of replicates for each experiment is shown in the figure captions. Comparisons among multiple groups were conducted using the one-way ANOVA followed by a Tukey test (*P* < 0.01).

### Accession numbers

The protein- and gene-specific sequences referenced in this study can be found in the Arabidopsis Genome Initiative or GenBank/EMBL databases under the following accession numbers: IAA8, At2g22670; MPK3, At3g45640; MPK4, At4g01370; MPK6, At2g43790; TIR1, At3g62980; IAA2, At3g23030; IAA7, At3g23050, IAA19, At3g15540; GH3.3, At2g23170; DAD1, At2g44810; AOS, At5g42650; AOC4, At1g13280; OPR3, At2g06050; VSP1, At5g24780; MYB21, At3g27810; MYB24, At5g40350; MYB108, At3g06490; bZIP28, At3g10800; bZIP60, At1g42990; and Tubulin, At5g62690.

## Supplementary Material

kiae470_Supplementary_Data

## Data Availability

The data underlying this article are available in the article and in its online supplementary material.

## Supplementary Data

**Supplementary Figure S1.** Heat stress inhibits auxin signaling via MPK cascade.

**Supplementary Figure S2.** An in vitro pull-down assay showing the interaction between IAA8 and MPKs.

**Supplementary Figure S3.** The LUC complementation imaging assay demonstrates the in planta interaction between EV and MPKs.

**Supplementary Figure S4.** In vitro phosphorylation of IAA8^WT^ by recombinant MPK3 and MPK4.

**Supplementary Figure S5.** Predicted amino acid sequence of IAA8.

**Supplementary Figure S6.** Identification of MPK6-phosphorylated peptides in GST-IAA8 via mass spectrometry.

**Supplementary Figure S7.** Generation of three independent IAA8^WT^ OX, IAA8^AAA^ OX, and IAA8^DDD^ OX plants.

**Supplementary Figure S8.** Phenotypes of Col-0, IAA8^WT^ OX, IAA8^AAA^ OX, and IAA8^DDD^ OX plants during the vegetative stage.

**Supplementary Figure S9.** Statistical analyses of the floral organ phenotypes in Col-0 and three independent IAA8^WT^ OX, IAA8^AAA^ OX, and IAA8^DDD^ OX plants.

**Supplementary Figure S10.** Comparison of the fold reduction in the length of flower organs among Col-0 and IAA8 transgenic plants under heat stress conditions.

**Supplementary Figure S11.** LUC complementation imaging assay to detect the interactions of IAA8^WT^, IAA8^AAA^, and IAA8^DDD^ with TIR1 in *N. benthamiana* leaves.

**Supplementary Figure S12.** Inhibition of the auxin response by IAA8^DDD^.

**Supplementary Figure S13.** Expression of auxin-responsive genes was down-regulated in IAA8^DDD^ OX plants.

**Supplementary Figure S14.** Transcript levels of JA biosynthesis-related genes and the amount of JA in Col-0 and IAA8 transgenic plants.

**Supplementary Figure S15.** Structural comparisons of IAA8^WT^, phosphorylated IAA8^WT^ (P-IAA8^WT^), and IAA8^DDD^, observed by a computer modeling analysis.

**Supplementary Figure S16.** Comparison of the stability between phospho-mimicking and gain-of-function (IAA8^P170L^) mutants of IAA8 protein using a cell-free protein degradation assay.

**Supplementary Table S1.** Identification of phosphopeptides by mass spectrometry via TiO_2_ chromatography from the tryptic digestion of IAA8 phosphorylated by MPK6.

**Supplementary Table S2.** Primers used for cloning and site-directed mutagenesis.

**Supplementary Table S3.** Primers used for RT-qPCR and ChIP-qPCR.

## Dive Curated Terms

The following phenotypic, genotypic, and functional terms are of significance to the work described in this paper:

IAA8 Gramene: AT2G22670
IAA8 Araport: AT2G22670

TIR1 Gramene: AT3G62980
TIR1 Araport: AT3G62980

IAA15 Gramene: AT1G80390
IAA15 Araport: AT1G80390

MPK12 Gramene: AT2G46070
MPK12 Araport: AT2G46070

IAA33 Gramene: AT5G57420
IAA33 Araport: AT5G57420

ibr5 Gramene: AT2G04550
ibr5 Araport: AT2G04550

TMK1 Gramene: AT1G66150
TMK1 Araport: AT1G66150

bZIP28 Gramene: AT3G10800
bZIP28 Araport: AT3G10800

MYB24 Gramene: AT5G40350
MYB24 Araport: AT5G40350

DAD1 Gramene: At2g44810
DAD1 Araport: At2g44810

IAA19 Gramene: AT3G15540
IAA19 Araport: AT3G15540

MPK4 Gramene: AT4G01370
MPK4 Araport: AT4G01370

auxin CHEBI: CHEBI:22676

MPK3 Gramene: AT3G45640
MPK3 Araport: AT3G45640

MPK6 Gramene: AT2G43790
MPK6 Araport: AT2G43790

bZIP60 Gramene: At1g42990
bZIP60 Araport: At1g42990
