# Supplementary material for: Phosphorylation of auxin signaling repressor IAA8 by heat-responsive MPKs causes defective flower development
Source: Plant Physiol. 2024 Sep 6;196(4):2825–40. doi: 10.1093/plphys/kiae470 (PMC11638004; doi:10.1093/plphys/kiae470)
Supplement: kiae470_Supplementary_Data [file kiae470_supplementary_data.zip › kiae470_Supplementary_Data.pdf]

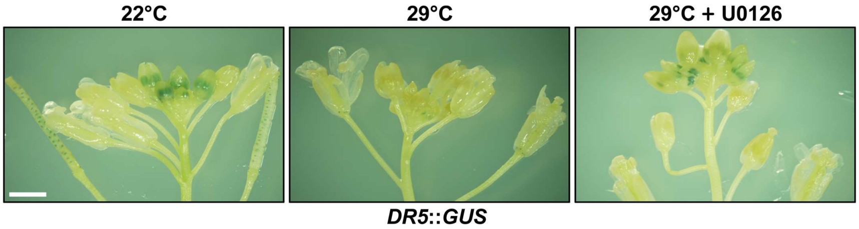

Supplementary Figure S1. Heat stress inhibits auxin signaling via MPK cascade. GUS activities were observed in the flowers of *DR5*::*GUS* plants grown under normal conditions (22°C), heat stress conditions (29°C), and heat stress conditions with U0126 (29°C + U0126). The scale bar represents 1 cm.

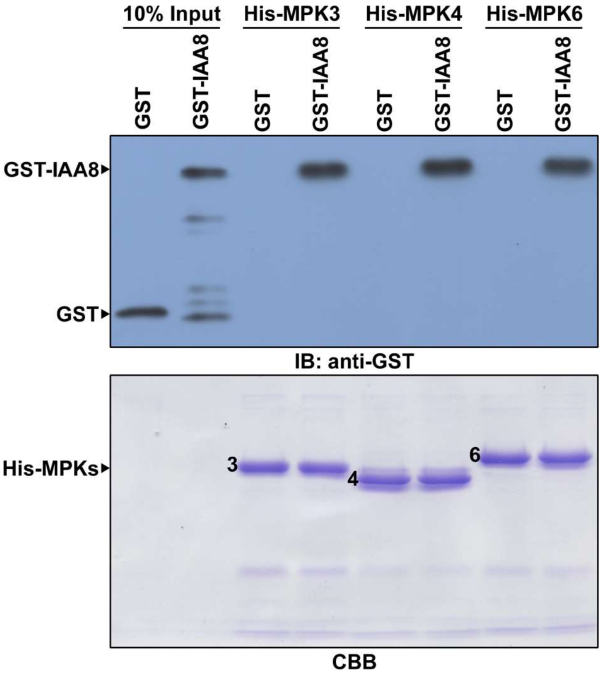

Supplementary Figure S2. An *in vitro* pull-down assay showing the interaction between IAA8 and MPKs. The interactions between GST-fused IAA8 and His-fused MPK3, MPK4, and MPK6 were tested. His-fused proteins were precipitated using Ni-NTA agarose resin. GST and GST-IAA8 were analyzed by immunoblotting using anti-GST antibodies (upper panel). The SDS–PAGE gel was stained by Coomassie Brilliant Blue (CBB) (lower panel). IB, Immunoblotting.

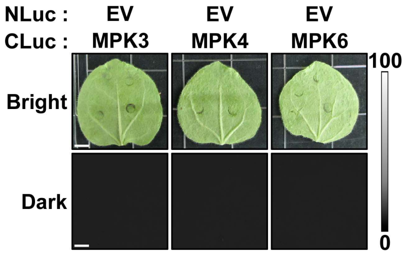

Supplementary Figure S3. The LUC complementation imaging assay demonstrates the *in planta* interaction between EV and MPKs. This result displays bright field (bright) and luminescence (dark) images of *N. benthamiana* leaves co-infiltrated with *NLuc* (*EV*) and *CLuc-MPKs*, serving as a negative control for Figure 1b. The scale bar represents 0.5 cm. The black-and-white color bar indicates the range of luminescence intensity. EV, Empty vector.

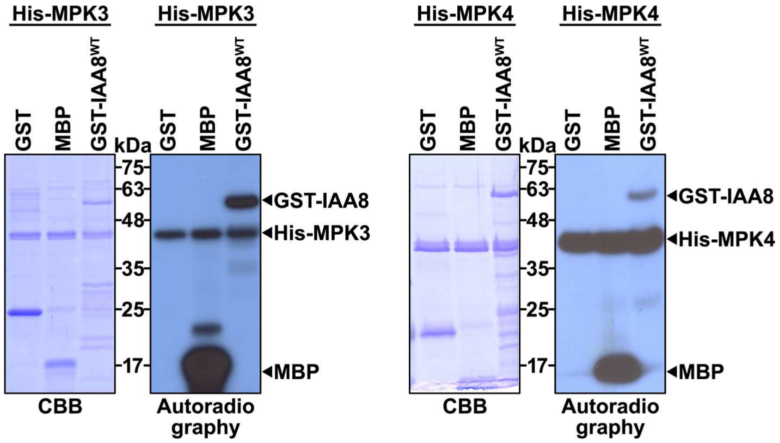

Supplementary Figure S4. *In vitro* phosphorylation of IAA8^{WT} by recombinant MPK3 and MPK4. The recombinant proteins were incubated in protein kinase buffer containing [γ-^{32}P] ATP. After incubation, the proteins were separated using 10% SDS–PAGE. Phosphorylated IAA8 was detected by autoradiography after gel electrophoresis (right panel). The recombinant proteins were visualized by Coomassie Brilliant Blue (CBB) staining (left panel). MBP and GST were used as positive and negative substrate control, respectively.

```
MSYRLLSVDK DELVT*SPCLK ERNYLGLSDC SSVDSSTIPN VVGKSNLNFK ATELRLGLPE     60
                                                          Domain I
SQ*SPERETDF GLL*SPR*TPDE KLLFPLLPSK DNGSATTGHK NVVSGN*KRGF ADTWDEFSGV    120

KGSVRPGGGI NMML*SPKVKD VSKSIQEERS HAKGGLNNAP AAKAQVVGWP PIRSYRKNTM    180
                                                       Domain II
ASSTSKNTDE VDGKPGLGVL FVKVSMDGAP YLRKVDLRTY TSYQQLSSAL EKMFSCFTLG    240
                          Domain III
QCGLHGAQGR ERMSEIKLKD LLHGSEFVLT YEDKDGDWML VGDVPWEIFT ETCQKLKIMK    300
                                      Domain IV
GSDSIGLAPG AVEKSKNKER V                                              321
```

* Putative phosphorylation sites

* Putative ubiquitination site

Supplementary Figure S5. Predicted amino acid sequence of IAA8. Four conserved domains of IAA8 are underlined. The red and blue asterisks indicate predicted phosphorylation and ubiquitination sites, respectively. The positions of amino acid residues are marked on the right.

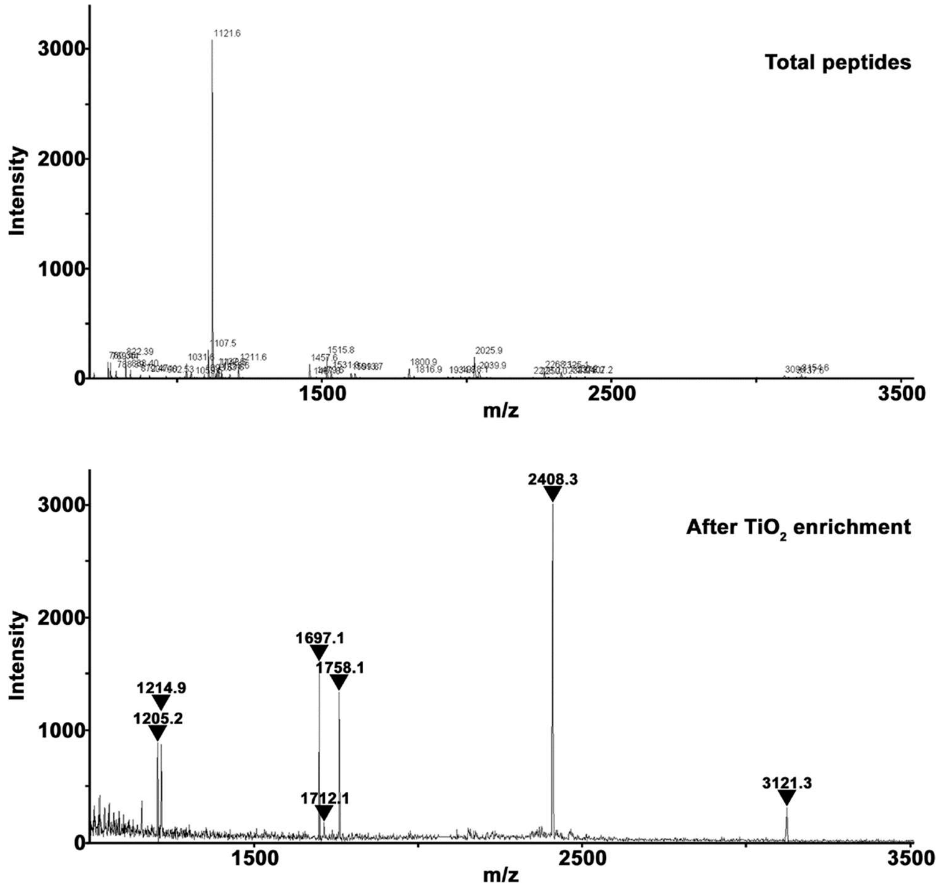

Supplementary Figure S6. Identification of MPK6-phosphorylated peptides in GST-IAA8 via mass spectrometry. The peptide mass fingerprints of MPK6-phosphorylated GST-IAA8, with and without TiO_2 purification after trypsin digestion, are shown. Seven phospho-peptide peaks (indicated by arrowheads) were detected from phosphorylated GST-IAA8.

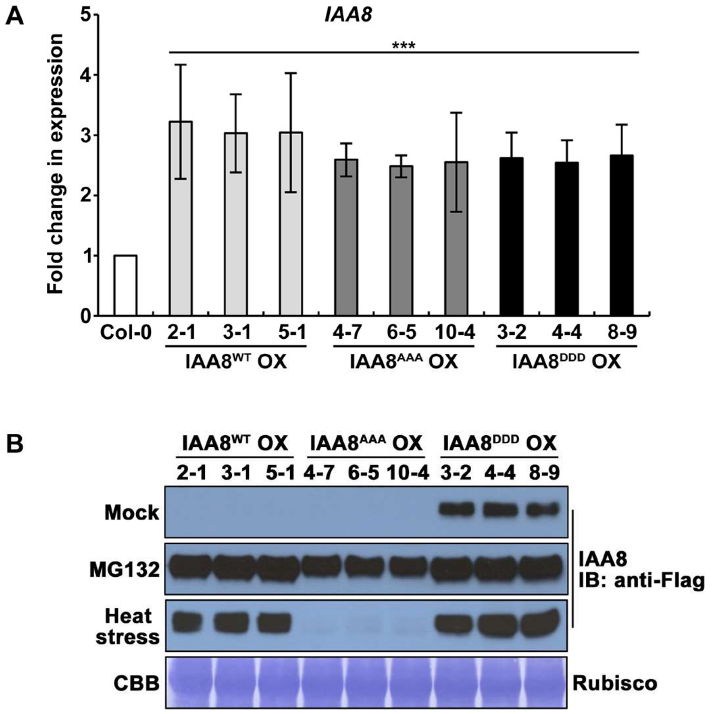

Supplementary Figure S7. Generation of three independent IAA8^{WT} OX, IAA8^{AAA} OX, and IAA8^{DDD} OX plants. (A) Transcript levels of *IAA8* in the IAA8 transgenic plant were measured by qPCR using gene-specific primers. The bars represent the mean ± S.D. ($n = 3$). Significant differences were determined by one-way ANOVA followed with a Tukey test (***, $P < 0.001$). **(B)** Protein levels of IAA8 in transgenic plants treated with mock, MG132, and heat stress were analyzed. IAA8 proteins were detected by immunoblotting with anti-Flag antibodies. The representative Coomassie Brilliant Blue (CBB) staining showed the Rubisco band as a loading control. IB, Immunoblotting.

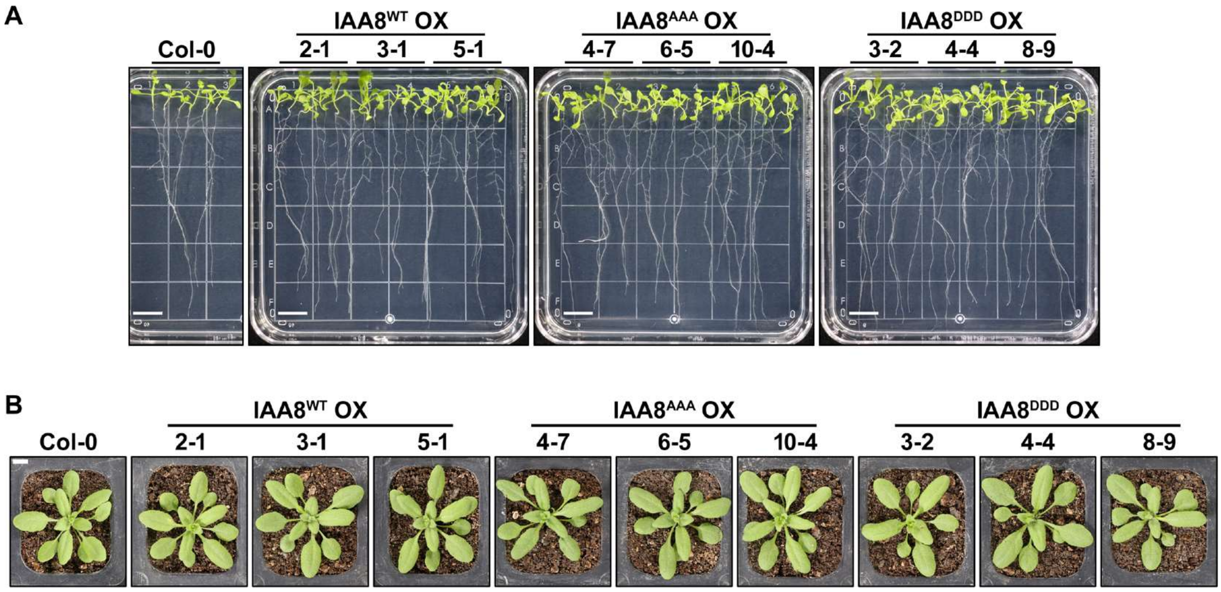

Supplementary Figure S8. Phenotypes of Col-0, IAA8^{WT} OX, IAA8^{AAA} OX, and IAA8^{DDD} OX plants during the vegetative stage. (A) Root growth phenotypes of Col-0 and three independent IAA8^{WT} OX, IAA8^{AAA} OX, and IAA8^{DDD} OX plants. 12-day-old plants grown vertically on MS plates were photographed. The scale bar represents 1 cm. **(B)** Rosette leaves phenotypes of 4-week-old Col-0, IAA8^{WT} OX, IAA8^{AAA} OX, and IAA8^{DDD} OX plants grown in soil. The scale bar represents 1 cm.

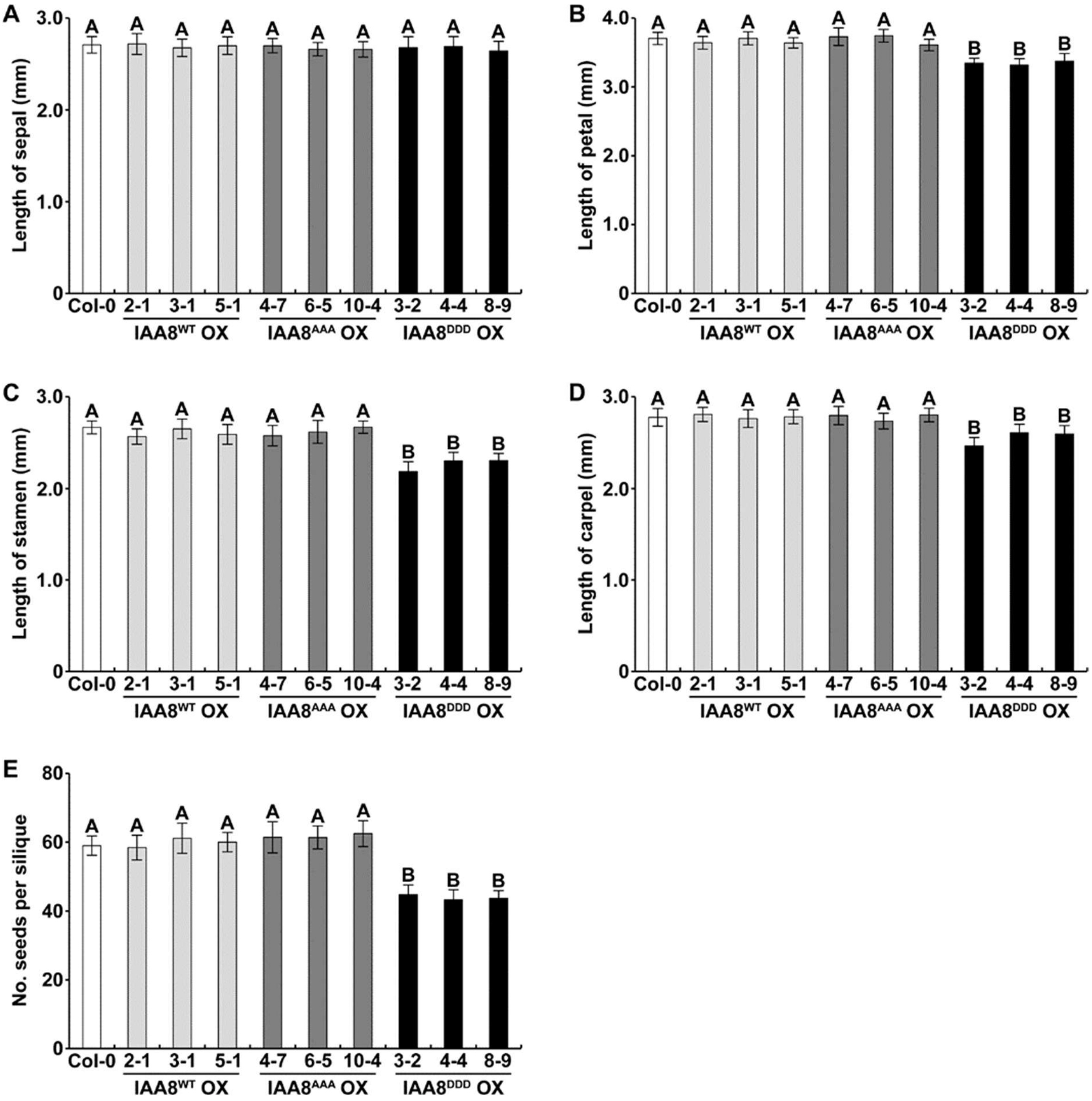

Supplementary Figure S9. Statistical analyses of the floral organ phenotypes in Col-0 and three independent IAA8^{WT} OX, IAA8^{AAA} OX, and IAA8^{DDD} OX plants. Lengths of sepals (A), petals (B), stamens (C), and carpels (D) were measured in mature flowers of adult plants (35-day-old). (E) The number of seeds were measured from mature siliques. The bars represent the mean ± S.D. ($n = 12$). Different letters on all graphs represent statistically significant differences between genotypes by one-way ANOVA followed by a Tukey test ($P < 0.01$).

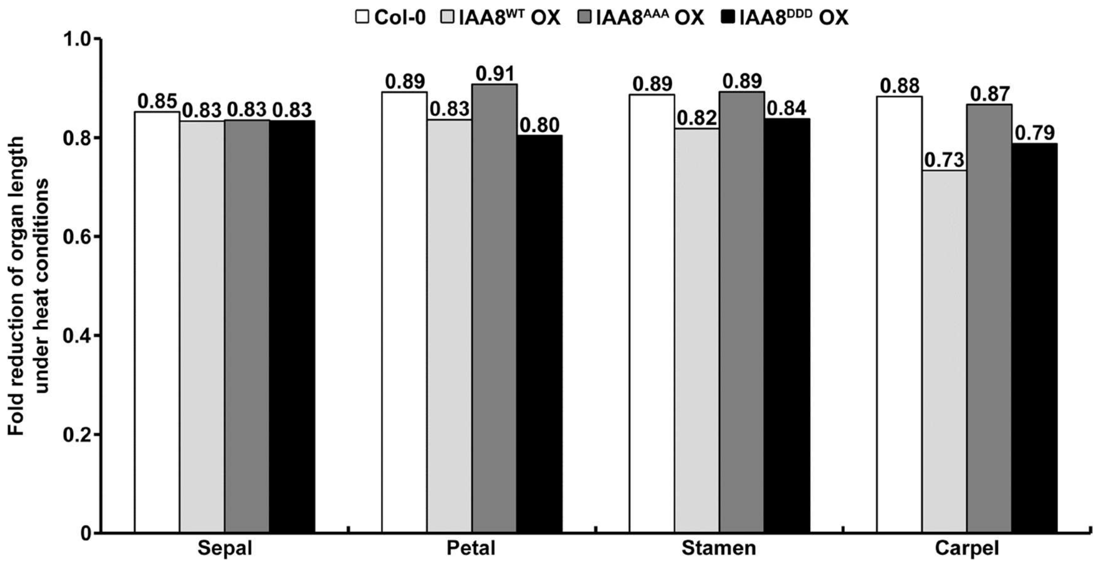

Supplementary Figure S10. Comparison of the fold reduction in the length of flower organs among Col-0 and IAA8 transgenic plants under heat stress conditions. The reduction in length of flower organs for each IAA8 plants under heat stress conditions, relative to normal conditions, is shown as the relative fold reduction in Figure 4.

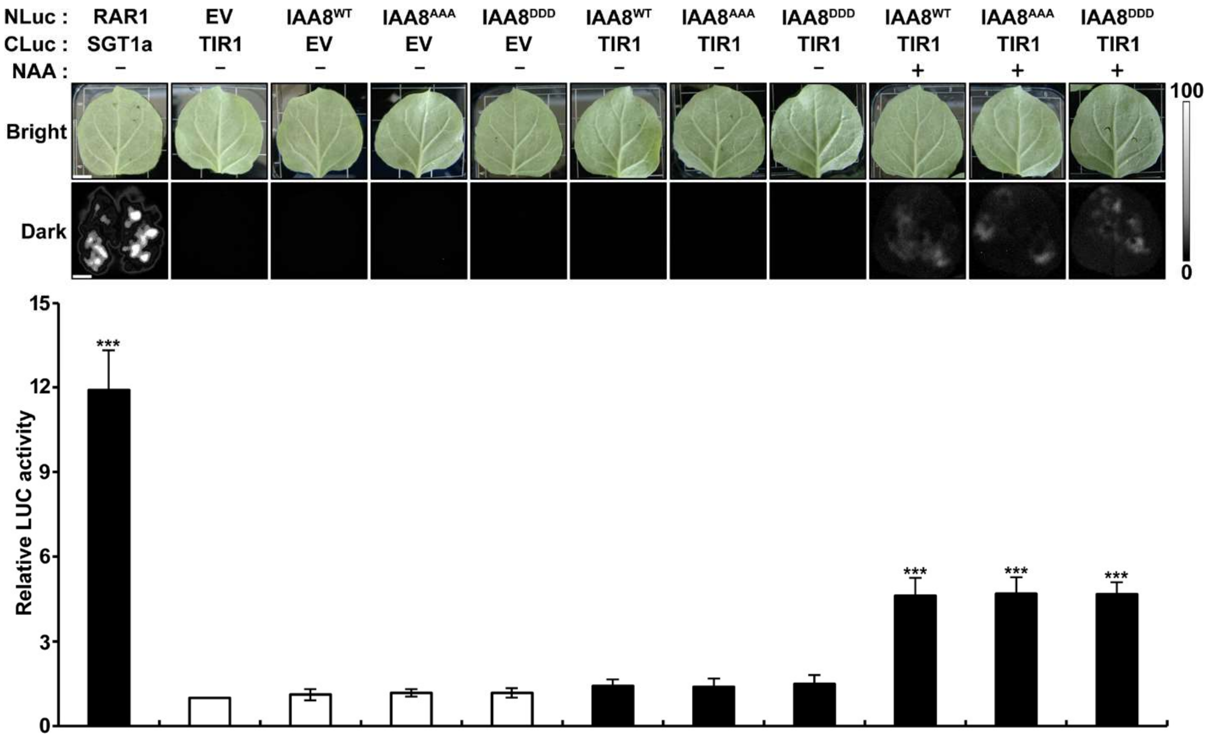

Supplementary Figure S11. LUC complementation imaging assay to detect the interactions of IAA8^{WT}, IAA8^{AAA}, and IAA8^{DDD} with TIR1 in *N. benthamiana* leaves. The upper panel displays bright-field (bright) and luminescence (dark) images of *N. benthamiana* leaves co-infiltrated with *Agrobacterium* strains containing the indicated combinations of *NLuc*- and *CLuc*-fusion constructs in the absence and presence of NAA. The *SGT1a-NLuc*/*CLuc-RAR1* combination was used as a positive control. EV refers to NLuc and CLuc empty vectors. The leaves were imaged 3 d after infiltration. The scale bar represents 0.5 cm. The black-and-white color bar indicates the range of luminescence intensity. The quantification of LUC activity in the leaves is presented in the lower panel. Luminescence intensities are expressed relative to those of leaves infiltrated with the *NLuc* (EV)/*CLuc-TIR1* combination. The bars represent the mean ± S.D. (n = 3). Significant differences were determined by one-way ANOVA followed by a Tukey test ($P < 0.001$). EV, Empty vector.

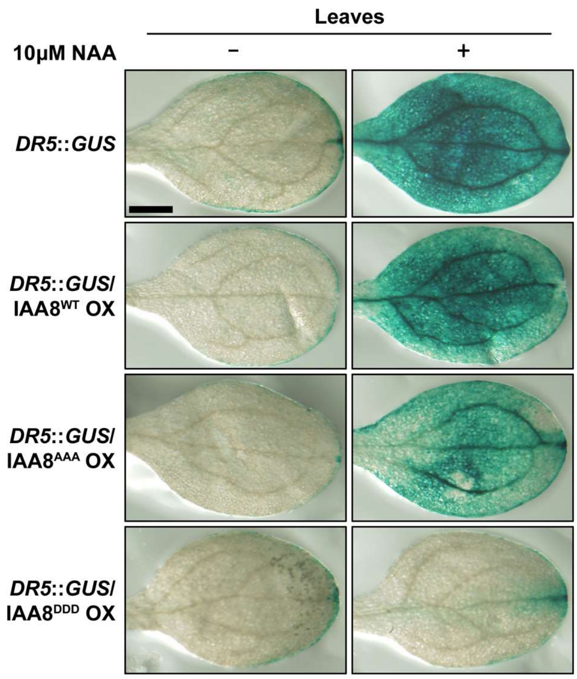

Supplementary Figure S12. Inhibition of the auxin response by IAA8^{DDD}. Two-weed-old *DR5*::*GUS*, *DR5*::*GUS*/IAA8^{WT} OX, *DR5*::*GUS*/IAA8^{AAA} OX, and *DR5*::*GUS*/IAA8^{DDD} plants grown on MS plates, were treated with or without NAA for 3 h. Subsequently, these seedlings were incubated in 5-bromo-4-chloro-3-indolyl glucuronide (X-gluc) for GUS staining for 12 h, and the leaves were photographed. The scale bar represents 1 cm. "–" and "+" indicate absence and presence, respectively.

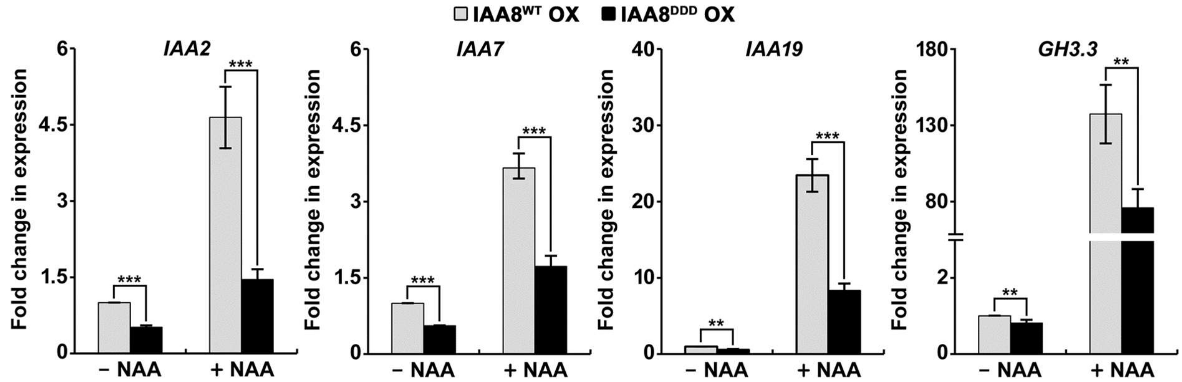

Supplementary Figure S13. Expression of auxin-responsive genes was down-regulated in IAA8^{DDD} OX plants. Total RNAs were extracted from IAA8^{WT} and IAA8^{DDD} OX plants treated with or without 10 μM NAA for 3 h. The transcript levels of *IAA2*, *IAA7*, *IAA19*, and *GH3.3* were measured by qPCR using gene-specific primers. The bars represent the mean ± S.D. (n = 3). Significant differences were determined by the Student's t-test (**, $P < 0.01$; ***, $P < 0.001$).

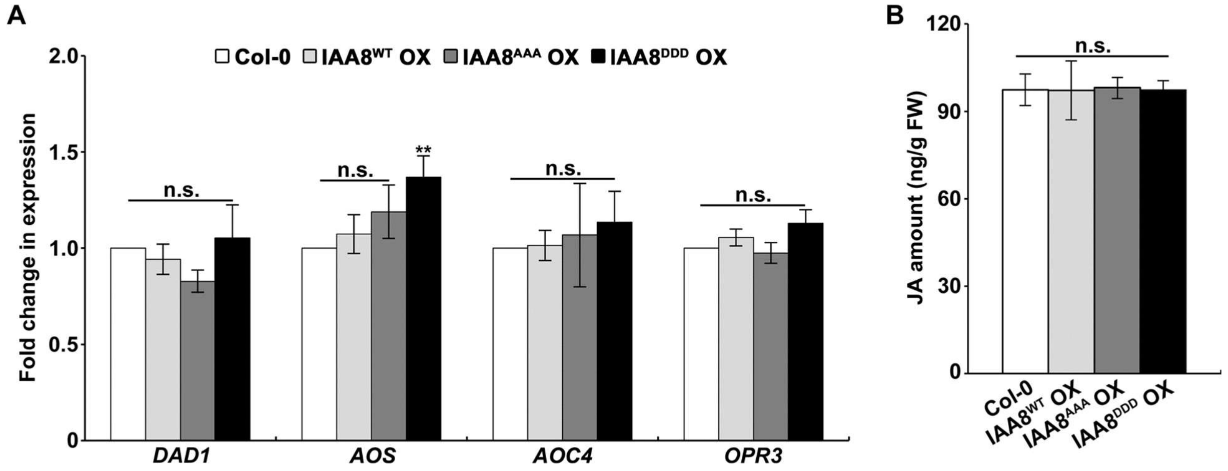

Supplementary Figure S14. Transcript levels of JA biosynthesis-related genes and the amount of JA in Col-0 and IAA8 transgenic plants. (A) The transcript levels of *DAD1*, *AOS*, *AOC4*, and *OPR3* were analyzed via qPCR. Total RNAs were extracted from flowers of Col-0 and IAA8 transgenic plants. qPCR analyses were performed using gene-specific primers. The bars represent the mean ± S.D. (*n* = 3). Significant differences were determined by one-way ANOVA followed by a Tukey test (**, P < 0.01). n.s., not significantly different (P > 0.05). **(B)** The amount of JA in flowers of Col-0 and IAA8 transgenic plants. The bars represent the mean ± S.D. (*n* = 3). Significant differences were determined by one-way ANOVA followed by a Tukey test. n.s., not significantly different (P > 0.05).

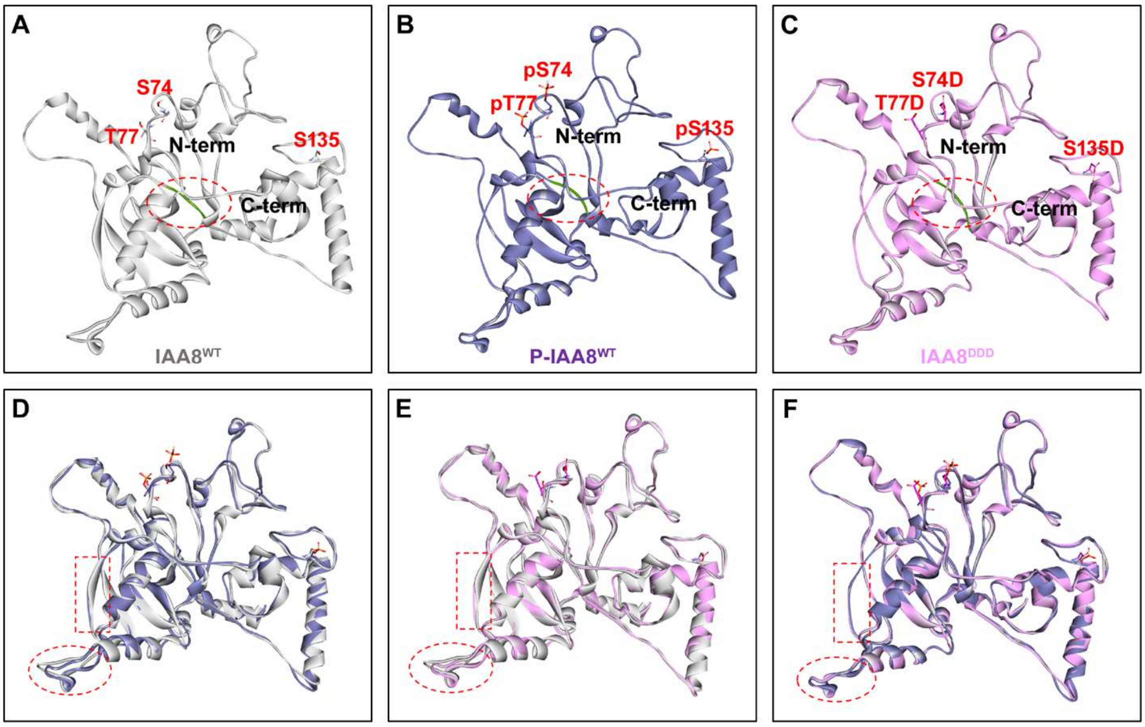

Supplementary Figure S15. Structural comparisons of IAA8^{WT}, phosphorylated IAA8^{WT} (P-IAA8^{WT}), and IAA8^{DDD}, observed by a computer modeling analysis. (A-C) Structures of the IAA8^{WT} (**A**, grey), P-IAA8^{WT} (**B**, purple), and IAA8^{DDD} (**C**, pink) are depicted. The phosphorylation sites are marked in red. The domain II of IAA8 is displayed as a green within dotted circles. The N-terminus and C-terminus of IAA8 are indicated as N-term and C-term, respectively. **(D-F)** Superimposed structures between IAA8 and its variants. IAA8^{WT} is superimposed with P-IAA8^{WT} **(D)** and IAA8^{DDD} **(E)**. P-IAA8^{WT} is superimposed with IAA8^{DDD} **(F)**. Conformation changes in the structures of IAA8 and its variants are indicated by red dotted circles and squares. Root-mean-square deviations (RMSD) were found to be 0.89 Å **(D)**, 0.89 Å **(E)**, and 0.62 Å **(F)**.

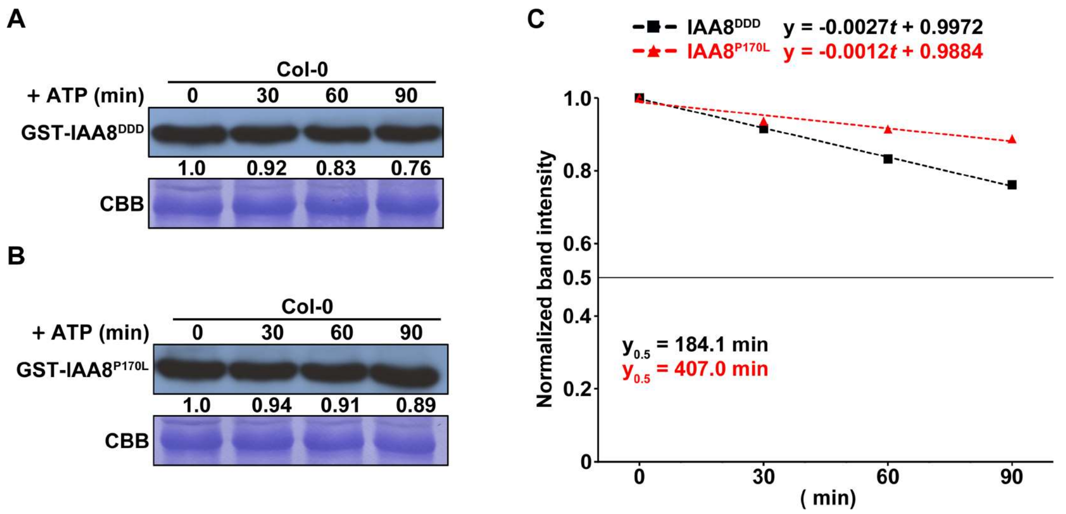

Supplementary Figure S16. Comparison of the stability between phospho-mimicking and gain-of-function (IAA8^{P170L}) mutants of IAA8 protein using a cell-free protein degradation assay. (A, B) Equal amounts of total proteins extracted from 2-week-old Col-0 plants were incubated with recombinant GST-IAA8^{DDD} **(A)** and GST-IAA8^{P170L} **(B)** proteins in the presence of ATP. IAA8 proteins were detected using anti-GST antibodies. The Coomassie Brilliant Blue (CBB) staining showed the Rubisco band as a loading control (lower panel). **(C)** Linear regressions of the immunoblot band intensities **(A, B)** measured by ImageJ represent the degradation rates of GST-IAA8. The line and value of $y_{0.5}$ indicate the half-life of GST-IAA8 proteins.

Supplementary Table S1. Identification of phospho-peptides by mass spectrometry via TiO_2 chromatography from the tryptic digestion of IAA8 phosphorylated by MPK6.

Phosphor-peptide sequence	Position of IAA8	No. phosphate groups	(M+H)		Putative phosphorylation site
			Expected	Measured	
VS^{205}MDGAPYLR + Oxidation	204-213	1	1203.6	1203.5	S205
ETDFGLLS^{74}PR	67-76	1	1213.9	1213.5	S74
GSVRPGGGINMMLS^{135}PK + Oxidation	122-137	1	1696.0	1695.8	S135
GSVRPGGGINMMLS^{135}PK + 2 Oxidation	122-137	1	1712.0	1711.8	S135
T^{77}PDEKLLFPLLPSK	77-90	1	1756.6	1756.8	T77
LGLPESQSPERETDFGLLS^{74}PR	56-76	1	2407.2	2407.1	S74
GFADTWDEFSGVKGSVRPGGGINMMLS^{135}PK	109-137	1	3119.2	3119.4	S135

Amino acid residue colored red that could potentially be phosphorylated by MPK6.

Supplementary Table S2. Primers used for cloning and site-directed mutagenesis.

Construct	Position	Sequence
IAA8^{WT}	F	5'- *ggatccATGTCTTATCGATTGCTAAG* -3'
	R	5'- *ctcgagTCAAACCCGCTCTTTGTT* -3'
$\text{IAA8}^{\text{S74A}}$	F	5'- *TTGCTGGCTCCGAGAACACCCGATGAGAAG* -3'
	R	5'- *TCTCGGAGCCAGCAAACCGAAATCAGTCTC* -3'
$\text{IAA8}^{\text{T77A}}$	F	5'- *CCGAGAGCACCCGATGAGAAGCTTCTCTTC* -3'
	R	5'- *ATCGGGTGCTCTCGGACTCAGCAAACCGAA* -3'
$\text{IAA8}^{\text{S135A}}$	F	5'- *ATGTTGGCGCCGAAAGTTAAGGATGTCTCG* -3'
	R	5'- *TTTCGGCGCCAACATCATGTTGATTCCTCC* -3'
$\text{IAA8}^{\text{S74D}}$	F	5'- *GGTTTGCTGGACCCGAGAACACCCGATGAG* -3'
	R	5'- *TGTTCTCGGGTCCAGCAAACCGAAATCAGT* -3'
$\text{IAA8}^{\text{T77D}}$	F	5'- *AGTCCGAGAGACCCCGATGAGAAGCTTCTC* -3'
	R	5'- *CTCATCGGGGTCTCTCGGACTCAGCAAACC* -3'
$\text{IAA8}^{\text{S135D}}$	F	5'- *ATGTTGGACCCGAAAGTTAAGGATGTCTCG* -3'
	R	5'- *TTTCGGGTCCAACATCATGTTGATTCCTCC* -3'
His-MPK3	F	5'- *ggatccATGAACACCGGCGGTGGCCA* -3'
	R	5'- *gtcgacCTAACCGTATGTTGGATTGA* -3'
His-MPK4	F	5'- *ggatccATGTCGGCGGAGAGTTGTTT* -3'
	R	5'- *gtcgacCTACACTGAGTCTTGAGGAT* -3'
His-MPK6	F	5'- *ggatccATGGACGGTGGTTCAGGTCA* -3'
	R	5'- *gtcgacCTATTGCTGATATTCTGGAT* -3'
IAA8 (yeast two-hybrid)	F	5'- *gaattcATGTCTTATCGATTGCTAAG* -3'
	R	5'- *ctcgagTCAAACCCGCTCTTTGTT* -3'
MPK3 (yeast two-hybrid)	F	5'-*ggatccgtATGAACACCGGCGGTGGCCA*-3'
	R	5'-*ctgcagCTAACCGTATGTTGGATTGA*-3'
MPK4 (yeast two-hybrid)	F	5'-*ggatccgtATGTCGGCGGAGAGTTGTTT*-3'
	R	5'-*ctgcagCTACACTGAGTCTTGAGGAT*-3'
MPK6 (yeast two-hybrid)	F	5'-*ggatccgtATGGACGGTGGTTCAGGTCA*-3'
	R	5'-*ctgcagCTATTGCTGATATTCTGGAT*-3'

Supplementary Table S3. Primers used for RT-qPCR and ChIP-qPCR.

Gene	Position	Sequence
IAA8	F	5'- *GGTCAATGTGGTCTTCATGG* -3'
	R	5'- *ACCTGGAGCTAAGCCAATAG* -3'
IAA2	F	5'- *CATGAAGGGATCCGACGCTC* -3'
	R	5'- *GTCGGACAAAACCCCGAAGT* -3'
IAA7	F	5'- *GGACGGTGCTCCATATCTGA* -3'
	R	5'- *CTTTGTCCTCGTAGCTTGGC* -3'
IAA19	F	5'- *GAGCATGGATGGTGTGCCTTAT* -3'
	R	5'- *TTCGCAGTTGTCACCATCTTTC* -3'
GH3.3	F	5'- *ATGGAGGAGTCGTTGAACTCTGTG* -3'
	R	5'- *AAGCTCCATTATTGGCGTGAAACTC* -3'
DAD1	F	5'- *GTCATCACCAAAGTTCCTGG* -3'
	R	5'- *TTCTCTGAATGGACACGTGG* -3'
AOS	F	5'- *CATGTGTTGTGGTCGAATGG* -3'
	R	5'- *GCTAGCTTTCCTTAACGACG* -3'
AOC4	F	5'- *CCAGAGAAGAAAGGTGATCG* -3'
	R	5'- *GGTAAATCAGCAGCAACACC* -3'
OPR3	F	5'- *GGCTATAGATCACTTGGACG* -3'
	R	5'- *CAGCTTGCATACCTAGTTCC* -3'
VSP1	F	5'- *ACACTCTCCTCTCTAGTATT* -3'
	R	5'- *TTCGATCCGTTTGGCCTGCG* -3'
MYB21	F	5'- *GGTAATTGATTGATCGGTGG* -3'
	R	5'- *CCCCAAACCTCTACAACGAG* -3'
MYB24	F	5'- *CGTATCAACATGCCAGCAAT* -3'
	R	5'- *CCAAAGATCATCGACGCTCC* -3'
MYB104	F	5'- *CAAACCCTAACCCGGAATAC* -3'
	R	5'- *CTGAGGTTACTCTGCTCTTG* -3'
bZIP28	F	5'- *TGATATTGCTCCTGGTGCTA* -3'
	R	5'- *TTCTCTTGCCGTGGGTAG* -3'
bZIP60	F	5'- *GAAGGAGACGATGATGCTGTGGCT* -3'
	R	5'- *AGCAGGGAACCCAACAGCAGACT* -3'
Tubulin	F	5'- *CCAACAACGTGAAATCGACAG* -3'
	R	5'- *TCTTGGTATTGCTGGTACTCT* -3'

bZIP28 P1	F	5'- *TGGTCACCCATTGGTTAGTC* -3'
	R	5'- *TGCATTCCAAAGATGTTGAG* -3'
bZIP60 P1	F	5'- *GAACCACGTCATTAGATCAC* -3'
	R	5'- *GCTTATCAACCGAACTTGTC* -3'
bZIP60 P2	F	5'- *GGACAAGAGATACGACCTCC* -3'
	R	5'- *TGCAATTTCAATTAGAAACG* -3'
bZIP60 P3	F	5'- *GAACCACGTCATTAGATCAC* -3'
	R	5'- *GCTTATCAACCGAACTTGTC* -3'
bZIP60 P4	F	5'- *ACAGTACACTCCATCACATC* -3'
	R	5'- *GCGACCAAGTAAATGTGTAG* -3'
