# Supplementary material for: Phosphorylation of auxin signaling repressor IAA8 by heat-responsive MPKs causes defective flower development
Source: Plant Physiol. 2024 Sep 6;196(4):2825–40. doi: 10.1093/plphys/kiae470 (PMC11638004; doi:10.1093/plphys/kiae470)
Supplement: kiae470_Supplementary_Data [file pp.01010.2024-s06.pdf › pp.01010.2024-s06.pdf]

Plant Physiology®

IAA8 phosphorylation by heat-responsive MPKs causes defective flowers by perturbated expressions of flower-related genes

Sun Ho Kim, Shah Hussain, Huyen Trang Pham, Ulhas Kadam, Sunghwa Bahk, Zakiyah Ramadany, Jeongwoo Lee, Young Hun Song, Kyun Oh Lee, Jong Chan Hong, and Woo Sik Chung

Review Timeline:

Submission Date:	04-Mar-2021
Editorial Decision:	29-Mar-2021
Resubmission Received:	28-Jun-2024
Editorial Decision:	04-Jul-2024
Revision Received:	11-Jul-2024
Accepted:	02-Aug-2024

29-Mar-2021

Prof. Dr. Woo Sik Chung
Division of Applied Life Science (BK21 Plus program), Plant Molecular Biology and Biotechnology Research Center,
Gyeongsang National University
Jinju 52828
Korea (South), Republic of

RE: IAA8 phosphorylation by heat-responsive MPKs causes defective flower by increasing the expression of MYB genes

Dear Dr. Woo Sik Chung:

Thank you for giving us the opportunity to review your manuscript for publication in the Plant Physiology. The reviewers have found the manuscript of interest, but have raised a number of concerns that will require your attention. As these concerns are of some gravity and will entail substantial changes to the manuscript, you are likely to need additional time to address them. I must therefore reject the manuscript in its present form. Nonetheless, if you are able to address the points raised in review, I would welcome your resubmission of a manuscript that incorporates your responses.

In particular, I direct your attention to the following comments for consideration in a resubmitted manuscript.

- You are proposing that IAA phosphorylation is involved in inhibition of flower development at high temperature. This is an interesting idea, however, you have not directly tested this. You should compare the flower phenotype of the WT and different mutants/transgenic lines grown at different temperatures. Please carefully consider the suggestions from reviewer 2.

- Mixing data from independent transgenic lines is a problem (see reviewer 1), please present data from independent lines.

- Please carefully explain the assay you have done on Figure 2B as the reviewers had a hard time understanding. I understood that these are in gel kinase assays using MBP, IAA8 and controls as proteins for the assay. If that is correct you can simply indicate this in response to the reviewer's comments.

- Please carefully respond to all comments from the reviewers

Please note that any resubmission will be treated as a new entry to the system and is likely to go out for review.

If you do have any queries or concerns, you are welcome to contact me in the first instance.

Thank you again for allowing Plant Physiology to review your work. We look forward to hearing from you soon.

Sincerely,

Editor, Plant Physiology

-- Reviewer comments:
Reviewer #1 (Comments for the Author):

"IAA8 phosphorylation by heat-responsive MPKs causes defective flower by increasing the expression of MYB genes" by Kim et al.

In this manuscript, the authors linked phosphorylation of IAA8 by MPKs to heat-induced flower development defects. They first showed that MPKs physically interact with IAA8 using in vitro pulldown assays and split LUC complementation assay. They further showed that IAA8 is phosphorylated by MPKs. They overexpressed three versions of IAA8: WT, AAA, and DDD. They showed that the constitutively phosphorylated form increased the stability of IAA8. It was concluded that "MPK-Aux/IAA module enables plants to alter their development in response to environmental conditions.

Line 209, " We selected three independent lines of homozygous plants presenting highly increased levels of IAA8 transcripts (Supplemental Figure S5A). For further studies, we mixed seeds from these three lines together and used the mixtures.". This is a strange way to conduct phenotypic evaluation of transgenic plants. The reason that we need to use at least two independent transgenic lines is to rule out the possibility that the phenotypes are caused by positional effects or loss-of-function of the gene by T-DNA insertion. Using mixtures of the transgenic lines would not rule out the off-target effects. This is very unfortunate,

because the way the experiments conducted made the results unreliable.

Figure 1a: a reciprocal pulldown experiment should be performed. Fix MPKs on Ni column and pulldown GST-IAA8.

Figure 2B: I am really confused by this figure (Perhaps I missed something here?). Based on the text, those bands are phosphorylated MBP or IAA8? But in the figure, those appeared to be MPKs, especially if you consider MPK3 and 6 have different size? I cannot draw the conclusion from this figure that IAA8 is phosphorylated by MPKs.

Figure 2C, middle panel, those bands appeared to be double-bands. Please explain.

Figure 3C, it has two Y-axis. Very confusion

Reviewer #2 (Comments for the Author):

This manuscript describes phosphorylation of IAA8 by MPK MAP kinases. They present results showing that several MPKs can interact with and phosphorylate IAA8. They then use a phosphoproteomics experiment to identify three phosphorylation sites, and make mutant versions with these three sites changed to alanine (not phosphorylated) or to apartate (phosphomimic). Plants overexpressing these or the wild-type IAA8 were used for further characterizations. They find that the phosphomimic version causes subtly decreased petal, stamen, and carpel growth, whereas the wild-type and unphosphorylatable versions had no effect. They then show that heat stress increases phosphorylation of the wild-type protein, and this correlates with greater stability (and lower ubiquitination) of the protein. The phosphomimic version of the protein is also more stable than the wild-type protein. Lastly, they show effects on gene expression that are consistent with reduced auxin response, as would be expected for a stabilized Aux/IAA protein. They also see increased MYB expression, which is odd (see below). Overall the model that MPKs phosphorylate IAA8 is quite interesting, and adds a new mechanism by which environmental conditions regulate auxin response. I have several suggestions for improvements:

Major points

They have not looked at the effect of high temperature on flower development, and the phenotypes in Figure 3 (at normal temperature) are very subtle. Why not test how these genotypes respond to high temperature? Related to this, in Figure 3C, at what stage of development were the organ lengths measured?

The increased expression of MYB21, MYB24, and MYB108 in the IAA8(DDD) phosphomimic-expressing line is odd, since those genes are normally induced downstream of auxin response, and contribute to organ growth, especially petals and stamens. The authors emphasize that overexpression of these genes causes flower defects, but should also discuss the loss-of-function mutant phenotypes. Perhaps they see overexpression because of compensating feedback responses.

Another example of Aux/IAA proteins mediating response to stresses is from the Estelle lab (doi: 10.1016/j.cub.2016.12.016), and could be discussed.

Minor points:

In Figure 5B, the CHX 6h time point quantitation may be mislabeled, as the number 0.83 does not seem to correspond to the darkness of the band. Several other bands in Figure 5C also seem to have labels that are not proportionate to the band intensity.

In Figure 6A, I cannot see the supposed difference in X-Gluc staining among the different genotypes. They should either take better pictures (perhaps dissect out stamens at a particular stage), or remove this figure. Since they hypothesize that IAA8 acts in organ growth, DR5 is not a particularly useful marker to study. The expression in the anther is reported to be in the endothecium layer and has little to do with organ elongation.

Figure 2B. Can they assay a mpk3 mpk6 double mutant or perhaps a mkk mutant that might lack phosphorylation altogether?

Figure 2C. It could be helpful to test the IAA8(AAA) mutant protein as a negative control in this assay.

lines 181-182, this seems to be written backwards, since you cannot do the assay until you have made the mutant.

Editor comments

Thank you for giving us the opportunity to review your manuscript for publication in the Plant Physiology. The reviewers have found the manuscript of interest, but have raised a number of concerns that will require your attention. As these concerns are of some gravity and will entail substantial changes to the manuscript, you are likely to need additional time to address them. I must therefore reject the manuscript in its present form. Nonetheless, if you are able to address the points raised in review, I would welcome your resubmission of a manuscript that incorporates your responses.

In particular, I direct your attention to the following comments for consideration in a resubmitted manuscript.

- You are proposing that IAA phosphorylation is involved in inhibition of flower development at high temperature. This is an interesting idea, however, you have not directly tested this. You should compare the flower phenotype of the WT and different mutants/transgenic lines grown at different temperatures. Please carefully consider the suggestions from reviewer 2.

<Response to the comment>

As suggested by the reviewer 2, to evaluate whether heat stress inhibits flower development through stabilizing IAA8 mediated by phosphorylation, we investigated the effects of heat stress on flower development in IAA8 transgenic plants. These results were included in Figure 4 and described on page 8, lines 223-231 of the revised manuscript.

- Mixing data from independent transgenic lines is a problem (see reviewer 1), please present data from independent lines.

<Response to the comment>

We generated and selected three independent lines of each construct and observed that these lines showed not only similar expression patterns of IAA8 transcripts and proteins (Supplemental Figures S7) but also comparable flower development (Supplemental Figures S8

and S9). Therefore, we believed that the flower phenotypes of each transgenic plant are due to the expression of IAA8, not a side effect of T-DNA integration. Since three independent transgenic lines of each genotype exhibited comparably high transcript levels and similar phenotypes (Supplemental Figures S7-S9), we chose IAA8^{WT} OX #2-1, IAA8^{AAA} OX #4-7, and IAA8^{DDD} OX #8-9 as representative lines. To address the editor’s and reviewer’s concerns, we reanalyzed the phenotypes and conduced Western blots and qPCR using these representative lines. These results are presented in Figures 3, 4, 5A, 6B-D, and 7, as well as Supplemental Figures S8, S9, and S10 of the revised manuscript. We have also described information about the representative lines on page 8 lines 219-222.

- *Please carefully explain the assay you have done on Figure 2B as the reviewers had a hard time understanding. I understood that these are in gel kinase assays using MBP, IAA8 and controls as proteins for the assay. If that is correct you can simply indicate this in response to the reviewer's comments.*

<Response to the comment>

To clarify and enhance understanding, we have added further details in Figure 2B and described them on page 7, lines 183-185 of the revised manuscript. Additionally, to more clearly demonstrate phosphorylation of IAA8 by MPKs, we performed a phos-tag mobility shift assay in the presence of protein phosphatase (CIAP) and MEK inhibitor (U0126). Please see Figure 2C and page 7, lines 186-194 in the revised manuscript.

- *Please carefully respond to all comments from the reviewers*

<Response to the comment>

Based on the comments and suggestions of the editor and reviewers, we have revised the manuscript and addressed each reviewer's comments. Furthermore, to infer a possible molecular mechanism explaining how phosphorylation inhibits polyubiquitination of IAA8, we compared the protein structures of IAA8^{WT}, phosphorylated IAA8^{WT} (P-IAA8^{WT}), and IAA8^{DDD}. We have proposed a possible mechanism based on the structural comparisons of IAA8 variant proteins through computer modeling in the revised manuscript. Please see Figure S15 and page 14, lines 397-403.

Additionally, to explain the different phenotypes between gain-of-function mutant and phosphor-mimicking mutant of IAA8, we performed a cell-free protein degradation assay and found that the degradation rate of recombinant GST-IAA8^{P170L} (a gain-of-function mutant of IAA8) is much slower than that of GST- IAA8^{DDD}. The new data showing the different protein stabilities of IAA8^{DDD} and IAA8^{P170L} were included and described in the revised manuscript. Please see Figure S16 and page 15, lines 432-444.

Reviewer comments:

Reviewer #1 (Comments for the Author):

"IAA8 phosphorylation by heat-responsive MPKs causes defective flower by increasing the expression of MYB genes" by Kim et al.

In this manuscript, the authors linked phosphorylation of IAA8 by MPKs to heat-induced flower development defects. They first showed that MPKs physically interact with IAA8 using in vitro pulldown assays and split LUC complementation assay. They further showed that IAA8 is phosphorylated by MPKs. They overexpressed three versions of IAA8: WT, AAA, and DDD. They showed that the constitutively phosphorylated form increased the stability of IAA8. It was concluded that "MPK-Aux/IAA module enables plants to alter their development in response to environmental conditions.

Line 209, " We selected three independent lines of homozygous plants presenting highly increased levels of IAA8 transcripts (Supplemental Figure S5A). For further studies, we mixed seeds from these three lines together and used the mixtures.". This is a strange way to conduct phenotypic evaluation of transgenic plants. The reason that we need to use at least two independent transgenic lines is to rule out the possibility that the phenotypes are caused by positional effects or loss-of-function of the gene by T-DNA insertion. Using mixtures of the transgenic lines would not rule out the off-target effects. This is very unfortunate, because the way the experiments conducted made the results unreliable.

<Response to the comment>

We agreed with the reviewer's comments. We generated and selected three independent lines of each construct and observed that these lines showed not only similar expression patterns of IAA8 transcripts and proteins (Supplemental Figures S7) but also comparable flower development (Supplemental Figures S8 and S9). Therefore, we believed that the flower phenotypes of each transgenic plant are due to the expression of IAA8, not a side effect of T-DNA integration. Since three independent transgenic lines of each genotype exhibited comparably high transcript levels and similar phenotypes (Supplemental Figures S7-S9), we chose IAA8^{WT} OX #2-1, IAA8^{AAA} OX #4-7, and IAA8^{DDD} OX #8-9 as representative lines. To address the editor's and reviewer's concerns, we reanalyzed the phenotypes and conduced

Western blots and qPCR using these representative lines. These results are presented in Figures 3, 4, 5A, 5C, 6C, and 7, as well as Supplemental Figures S8, S9, and S10 of the revised manuscript. We have also described information about the representative lines on page 8 lines 219-222.

Figure 1a: a reciprocal pulldown experiment should be performed. Fix MPKs on Ni column and pulldown GST-IAA8.

<Response to the comment>

As suggested by the reviewer, we performed a reversed pull-down assay including an SDS-PAGE gel stained with Coomassie Brilliant Blue (CBB). These results are included in Figure 1A and Supplemental Figure S2 and are described on page 5, line 144 of the revised manuscript.

Figure 2B: I am really confused by this figure (Perhaps I missed something here?). Based on the text, those bands are phosphorylated MBP or IAA8? But in the figure, those appeared to be MPKs, especially if you consider MPK3 and 6 have different size? I cannot draw the conclusion from this figure that IAA8 is phosphorylated by MPKs.

<Response to the comment>

We understand he reviewer's misunderstanding. Most results from the *in-gel* kinase assays are presented like Figure 2B. In *in-gel* kinase assays, target substrates are embedded in gels and the phosphorylations are detected at the size of kinases. Therefore, in Figure 2B, the arrowheads at the positions of MPK3 and MPK6 indicate the phosphorylated bands of IAA8 by MPK3 and MPK6. The phosphorylated bands in MBP- or IAA8^{WT}-embedded gel were detected at the positions of MPK3 and MPK6 in Col-0, *mpk3*, and *mpk6* plants. However, these bands were not observed at those positions in GST-IAA8^{AAA}- or GST-embedded gels. These results conclude that native plant MPKs can phosphorylate IAA8. To clarify and enhance understanding, we have added further details in Figure 2B and described them on page 6, line 180 to page 7, line 181, and page 7, lines 182-185 of the revised manuscript. Additionally, to more clearly demonstrate phosphorylation of IAA8 by MPKs, we performed a phos-tag mobility shift assay in the presence of protein phosphatase (CIAP) and MEK inhibitor (U0126). Please see Figure 2C and page 7, lines 186-194 of the revised manuscript.

Figure 3C, it has two Y-axis. Very confusion

<Response to the comment>

We agree with the reviewer’s comments. We have separated the results for floral organ lengths and seed numbers in Figure 3E of the revised manuscript.

Reviewer #2 (Comments for the Author):

This manuscript describes phosphorylation of IAA8 by MPK MAP kinases. They present results showing that several MPKs can interact with and phosphorylate IAA8. They then use a phosphoproteomics experiment to identify three phosphorylation sites, and make mutant versions with these three sites changed to alanine (not phosphorylated) or to Aspartate (phosphomimic). Plants overexpressing these or the wild-type IAA8 were used for further characterizations. They find that the phosphomimic version causes subtly decreased petal, stamen, and carpel growth, whereas the wild-type and unphosphorylatable versions had no effect. They then show that heat stress increases phosphorylation of the wild-type protein, and this correlates with greater stability (and lower ubiquitination) of the protein. The phosphomimic version of the protein is also more stable than the wild-type protein. Lastly, they show effects on gene expression that are consistent with reduced auxin response, as would be expected for a stabilized Aux/IAA protein. They also see increased MYB expression, which is odd (see below). Overall the model that MPKs phosphorylate IAA8 is quite interesting, and adds a new mechanism by which environmental conditions regulate auxin response. I have several suggestions for improvements:

Major points

(A) They have not looked at the effect of high temperature on flower development, and the phenotypes in Figure 3 (at normal temperature) are very subtle. Why not test how these genotypes respond to high temperature? (B) Related to this, in Figure 3C, at what stage of development were the organ lengths measured?

<Response to the comment>

(A) We would like to thank you for the constructive suggestion. To evaluate whether heat stress inhibits flower development through stabilization of IAA8 mediated by phosphorylation, we investigated the effects of heat stress on flower development in IAA8 transgenic plants. These results are included in Figure 4 and described on page 8, lines 223-231 of the revised manuscript.

(B) The organ lengths were measured from the main inflorescence stems of 35-day-old plants.

The increased expression of MYB21, MYB24, and MYB108 in the IAA8(DDD) phosphomimic-expressing line is odd, since those genes are normally induced downstream of auxin response, and contribute to organ growth, especially petals and stamens. The authors emphasize that overexpression of these genes causes flower defects, but should also discuss the loss-of-function mutant phenotypes. Perhaps they see overexpression because of compensating feedback responses.

<Response to the comment>

We would like to thank you for the constructive comment. We already described that the loss-of-function mutants of MYB genes exhibited abnormal flower development at the page 3, line 89 to page 4, line 91 and page 11, lines 327-329 in the original manuscript. Therefore, we expected that *MYB* genes would be down-regulated by stabilizing IAA8 in IAA8^{DDD} OX plants compared to other plants. However, contrary to our expectation, the transcript levels of these genes were up-regulated in IAA8^{DDD} OX plants. Nevertheless, because transgenic plants overexpressing *MYB21* and *MYB24* also exhibited an abnormal flower phenotype (Song et al., 2011), we'd like to argue that the transcriptional increases of *MYB* genes cause the flower defective phenotype in IAA8^{DDD} OX plants. Moreover, we included new data showing the down-regulations of two membrane-associated transcription factors, *bZIP28* and *bZIP60*, in IAA8^{DDD} OX plants. These genes play positive roles in flower development under heat stress (Zhang et al., 2017). Furthermore, we revealed that the stabilized IAA8 negatively regulates *bZIP* gene expressions through direct association with promoters, as shown by ChIP assay. Therefore, we concluded that IAA8^{DDD} OX plants exhibited defective flower development caused by transcriptional perturbation of *bZIP* and *MYB* genes. These results are included in Figure 7 and described in the revised manuscript. Please see the title, page 2, lines 43-44, page 3, lines 82-87, page 11, lines 312-325, and page 14, lines 415-418 in the revised manuscript.

Another example of Aux/IAA proteins mediating response to stresses is from the Estelle lab (doi: 10.1016/j.cub.2016.12.016), and could be discussed.

<Response to the comment>

As suggested by the reviewer, we have discussed the key reference for Aux/IAA-mediated regulation of auxin signaling in response to stresses in the revised manuscript. Please see page 13, lines 364-366 and page 33, lines 973-976.

Minor points:

In Figure 5B, the CHX 6h time point quantitation may be mislabeled, as the number 0.83 does not seem to correspond to the darkness of the band. Several other bands in Figure 5C also seem to have labels that are not proportionate to the band intensity.

<Response to the comment>

We thank you for pointing out the mislabeling of band intensity in Figure 6B. As the reviewer mentioned, we have remeasured and relabeled the band intensity of Figures 6B and 6D in the revised manuscript. In addition, we have included data showing the stability of IAA8 in IAA8^{AAA} OX plants both in the absence and presence of auxin. Please see Figures 6C and page 10, lines 275-282 in the revised manuscripts.

In Figure 6A, I cannot see the supposed difference in X-Gluc staining among the different genotypes. They should either take better pictures (perhaps dissect out stamens at a particular stage), or remove this figure. Since they hypothesize that IAA8 acts in organ growth, DR5 is not a particularly useful marker to study. The expression in the anther is reported to be in the endothecium layer and has little to do with organ elongation.

<Response to the comment>

As suggested by the reviewer, we analyzed the GUS activities in *Arabidopsis* leaves and observed that the GUS activities were lower in IAA8^{DDD} OX plants than in other plants after treatment with NAA. Therefore, we have replaced the GUS activities of floral organs with

those of leaves and moved the original Figure 6A to Supplemental Figure S12 in the revised manuscript. Please see page 10, lines 287-291.

Figure 2B. Can they assay a mpk3 mpk6 double mutant or perhaps a mkk mutant that might lack phosphorylation altogether?

<Response to the comment>

It is infeasible to confirm the phosphorylation of IAA8 by native MPKs using the *mkk* mutants due to the complexity of the MKK-MPK networks. To use the *mpk3*/*mpk6* double mutant as suggested by the reviewer, we initially tested MPK activities in an estradiol-inducible *mpk3*/*mpk6* double mutant, in which *mpk3* is silenced with an estradiol-inducible *MPK3*-RNAi construct in a null *mpk6* mutant background (Cheng et al., 2015), using immunoblotting with anti-p42/p44, anti-MPK3, and anto-MPK6 antibodies. Unfortunately, MPK3 proteins were not suppressed by treatment with estradiol; thus, MPK3 activity was still detected when cotreatment with estradiol and stress in *mpk3*/*mpk6* mutant (see Figure 1 below). Consequently, we could not confirm the phosphorylation of IAA8 by MPK3 and MPK6 using the *mpk3*/*mpk6* mutant.

In this study, we observed that the phosphorylated bands corresponding to either MPK3 or MPK6 in Col-0 disappeared in *mpk3* and *mpk6* plants, respectively. This result suggests that IAA8 is phosphorylated by native plant MPK3 and MPK6. Additionally, we performed alternative experiments to more clearly demonstrate phosphorylation of IAA8 by MPKs. First, we performed a phos-tag mobility shift assay in the presence of protein phosphatase (CIAP) and MEK inhibitor (U0126). Second, we examined the stability of GST-IAA8 protein in Col-0, *mpk3*, and *mpk6* plants using a cell-free protein degradation assay. These results are included in Figures 2C and 5D, and described on page 7, lines 186-194 and page 9, lines 252-257 in the revised manuscript.

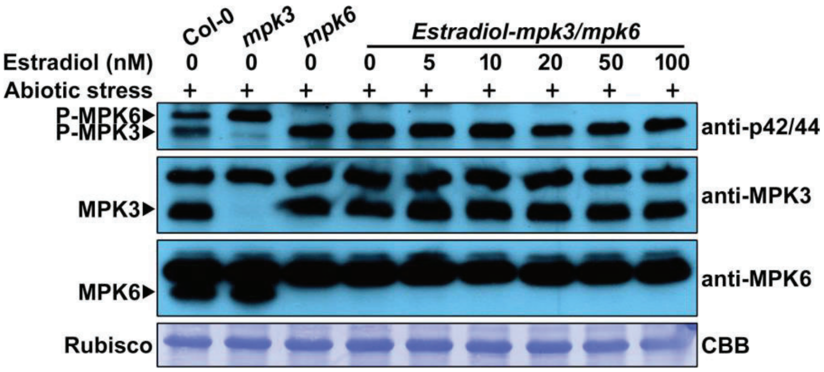

Figure 1. The protein levels and the activities of MPK3 and MPK6 after the co-treatment with estradiol and abiotic stress in Col-0, *mpk3*, *mpk6*, and *mpk3*/*mpk6* plants.

Figure 2C. It could be helpful to test the IAA8(AAA) mutant protein as a negative control in this assay.

<Response to the comment>

We appreciate the reviewer's constructive comment. Since the IAA8^{AAA} protein is unstable and could not be detected even after treatment with heat stress, we excluded the IAA8^{AAA} OX plants as a negative control in the phos-tag assay. To address the reviewer's concerns, we added the phos-tag mobility shift assay in the presence of protein phosphatase (CIAP) and MEK inhibitor (U0126). Please see Figure 2C and page 7, lines 186-194 in the revised manuscripts.

lines 181-182, this seems to be written backwards, since you cannot do the assay until you have made the mutant.

<Response to the comment>

We appreciate the reviewer's constructive comment. We did not describe the construction of the IAA8^{AAA} mutant in the section mentioned by the reviewer. This information has been clearly described in the revised manuscript. Please see page 6, lines 172-175.

Reference

Cheng Z, Li JF, Niu Y, Zhang XC, Woody OZ, Xiong Y, Djonović S, Millet Y, Bush J,

McConkey BJ, Sheen J, Ausubel FM (2015) Pathogen-secreted proteases activate a novel plant immune pathway. Nature **521**. 213–216

Reeves, P.H., Ellis, C.M., Ploense, S.E., Wu, M.F., Yadav, V., Tholl, D., Chételat, A., Haupt, I., Kennerley, B.J., Hodgens, C., Farmer, E.E., Nagpal, P., and Reed, J.W. (2012) A Regulatory Network for Coordinated Flower Maturation PLoS Genet. **8**, e1002506.

Zhang, S.S., Yang, H., Ding, L., Song, Z.T., Ma, H., Chang, F., and Liu, J.X. (2017) Tissue-Specific Transcriptomics Reveals an Important Role of the Unfolded Protein Response in Maintaining Fertility upon Heat Stress in Arabidopsis. Plant Cell **29**, 1007–1023.

04-Jul-2024

Prof. Woo Sik Chung
Gyeongsang National University
Jinju 52828
Korea (South), Republic of

RE: IAA8 phosphorylation by heat-responsive MPKs causes defective flowers by perturbated expressions of flower-related genes

Dear Dr. Woo Sik Chung:

We are pleased to accept your manuscript for publication in the Plant Physiology. This acceptance is contingent on revision based on the comments of our reviewers. In particular, please consider the following:

Reviewer 2 makes a number of interesting comments and has a few questions regarding your submission. I believe that these comments can all be addressed with text changes unless you think that some additional data you may have could be helpful.

Please highlight all changes and include a detailed annotation to changes to the text, with line numbers, and noting your responses to the comments.

To submit your revised manuscript, click:

Link Not Available

If you cannot return the revised manuscript within 8 weeks of receipt, please let us know. Otherwise, we will assume that you have elected not to revise the manuscript and are withdrawing it.

Thank you for allowing us to review your work. We look forward to hearing from you soon.

Sincerely,

Editor, Plant Physiology

-- Reviewer comments:
Reviewer #1 (Comments for the Author):

The authors have addressed my concerns.

Reviewer #2 (Comments for the Author):

The work shows a new mechanism by which an Aux/IAA protein stability may be regulated, through phosphorylation by MAP kinases. This causes subtle changes in flower organ lengths. They have addressed several previous comments. I now have the following suggestions:

In Figures 3 and 4, at what flower stage were the organ measurements done? The newly opened flowers have been assigned as stage 13 by Smyth et al.

In Figure 6B,C,D, it seems strange that MG132 was used in the protein turnover time courses. Is this correct? MG132 should have prevented the protein turnover as shown in Figure 5, unless it was washed out at the start of the turnover assays. Please clarify this point.

In Figure S15, it does not actually appear that the phosphorylations would cause much predicted conformational change to the protein backbone. Perhaps the actual effect is due mainly to changes in the side chains (size or charge). In this figure, it could be helpful to indicate where domain II (recognized by TIR1) is. Also, are the phosphorylation sites conserved in other Aux/IAA proteins?

They may wish to compare the flower phenotypes they see with those of the msg2/iaa19 gain-of-function mutant (see Tashiro et al. 2008, Physiologia Plantarum 137: 175-187).

In line 440 and other places (including the model figure 8), they seem to wish to conclude that the observed changes in expression of bZIP and MYB transcription factor genes cause the altered flower growth. However, as they have not tested this functionally, these are really just speculations, and the language could be toned down.

Reviewer #2 (Comments for the Author):

The work shows a new mechanism by which an Aux/IAA protein stability may be regulated, through phosphorylation by MAP kinases. This causes subtle changes in flower organ lengths. They have addressed several previous comments. I now have the following suggestions:

In Figures 3 and 4, at what flower stage were the organ measurements done? The newly opened flowers have been assigned as stage 13 by Smyth et al.

<Response to comment>

We measured the flower organs using the flower stage 14 of 35-day-old plants. We have included the flower developmental stage in the legends of Figures 3 and 4 of the revised manuscript.

In Figure 6B,C,D, it seems strange that MG132 was used in the protein turnover time courses. Is this correct? MG132 should have prevented the protein turnover as shown in Figure 5, unless it was washed out at the start of the turnover assays. Please clarify this point.

<Response to comment>

We appreciate the feedback from the reviewer. The plants pre-treated with MG132 were washed in water and then treated with NAA and CHX. We have clarified this experimental process in the revised manuscript as suggested. Please see page 10, lines 278-280; page 20, lines 582-584; and the legend of Figure 6.

In Figure S15, it does not actually appear that the phosphorylations would cause much predicted conformational change to the protein backbone. Perhaps the actual effect is due mainly to changes in the side chains (size or charge).

(A) In this figure, it could be helpful to indicate where domain II (recognized by TIR1) is.

(B) Also, are the phosphorylation sites conserved in other Aux/IAA proteins?

<Response to comment>

(A) As suggested by the reviewer, we have included the position of domain II in IAA8 in Figure S15.

(B) Previously, we reported that the predicted phosphorylation sites (SP and TP) of 9 canonical IAA proteins by MPK3 were not conserved (Kim et al., 2022). As suggested, we have briefly described this again in the revised manuscript. Please see page 14, lines 397-399.

They may wish to compare the flower phenotypes they see with those of the msg2/iaa19 gain-of-function mutant (see Tashiro et al. 2008, Physiologia Plantarum 137: 175-187).

<Response to comment>

As suggested by the reviewer, we have included the flower phenotypes for *msg2*/*iaa19* gain-of-function mutant in the revised manuscript. Please see page 3, lines 80-83, and page 34, lines 998-1000.

In line 440 and other places (including the model figure 8), they seem to wish to conclude that the observed changes in expression of bZIP and MYB transcription factor genes cause the altered flower growth. However, as they have not tested this functionally, these are really just speculations, and the language could be toned down.

<Response to comment>

We agree with the reviewer's comment. We have toned down the sentence in the revised manuscript. Please see page 14, line 415; page 15, lines 423-426; and the legend of Figure 8.

02-Aug-2024
Prof. Woo Sik Chung
Gyeongsang National University
Jinju 52828
Korea (South), Republic of

MSID: PP2024-RA-01010DR1
MS TITLE: IAA8 phosphorylation by heat-responsive MPKs causes defective flowers by perturbated expressions of flower-related genes

Dear Dr. Woo Sik Chung:

I am pleased to inform you that your revised manuscript "IAA8 phosphorylation by heat-responsive MPKs causes defective flowers by perturbated expressions of flower-related genes" has been accepted for publication in Plant Physiology. Your manuscript will be checked for consistency with journal formatting and image standards by one of our Science Editors, who will contact you shortly. They may have a number of queries and suggestions for you. Please keep in mind that they work to increase the online visibility of your article and its attraction for readers from the widest of backgrounds. You will have the opportunity to review any revisions, and I hope you will work constructively with them to get the most out of your research. You will also have the opportunity to review all changes when you receive the author proofs for your article.

Once validated for publication, your article will be sent to a Science Editor for a final review of your manuscript and source files. After your final files are uploaded, your article will appear in the next available issue and the online Preview version of your article will be posted after your signed Copyright and Checklist forms and final source files are received.

Please be sure to include the following in your manuscript file:
Harvard style citations
List of all author contributions and funding information at the beginning of manuscript after the titles and author list.
List all supplemental materials associated with the article, with titles, at the end of the ms, before the references and acknowledgments section.
Complete Figure Legends for all figures appearing in the article listed before the Literature Cited.

Thank you and congratulations on your Plant Physiology paper!

Sincerely,
Editor, Plant Physiology

=====
FIRST AUTHOR PROFILES
=====

We recognize the hard work that goes into being First Author of a Plant Physiology paper by publishing a "First Author Feature" for each First Author alongside your paper.

If the first author(s) would like to submit information for a feature on Facebook and Twitter, fill out the form by clicking on the link below:

Plant Physiology First Author Feature Form: https://forms.office.com/r/ZBCGZfhm8F
If you cannot access the form through the link above, please contact Rachel at rbelsky@aspb.org.

To upload an image of yourself to accompany your profile, please put your photo in the correct journal folder at the link below. You must label your image: Author First Name Last Name Article Title.

First Author Profile Image Dropbox: https://www.dropbox.com/scl/fo/xz029p7qjr2jml2rp2kmq/h?dl=0&rlkey=6fkxcaqmxx35ap152b2osy1fz

=====

IMPORTANT REMINDER: PEER REVIEW REPORTS
=====

If you opted to publish a peer review report along with your article during the original submission process, it will be prepared by the editorial staff and publicly posted with your manuscript, inside the zip file that contains any other supplemental material. As a reminder, the peer review report is a public record of all comments from editors and reviewers, as well as your prior responses, as you received them in the decision letters for each draft of your manuscript. If you agreed to publish this report and have changed your mind, or are not sure if you selected this option, please contact the editorial office as soon as possible before signing the license agreement from our publisher.

-- Reviewer comments:
